# Hierarchical motion perception as causal inference

**DOI:** 10.1038/s41467-025-58797-0

**Published:** 2025-04-24

**Authors:** Sabyasachi Shivkumar, Gregory C. DeAngelis, Ralf M. Haefner

**Affiliations:** 1 Brain and Cognitive Sciences, University of Rochester, Rochester, NY USA; 2 Zuckerman Mind Brain Behavior Institute, Columbia University, New York, NY USA; 3 Center for Visual Science, University of Rochester, Rochester, NY USA

**Keywords:** Computational models, Motion detection, Perception

## Abstract

Motion can only be defined relative to a reference frame; yet it remains unclear which reference frame guides perception. A century of psychophysical studies has produced conflicting evidence: retinotopic, egocentric, world-centric, or even object-centric. We introduce a hierarchical Bayesian model mapping retinal velocities to perceived velocities. Our model mirrors the structure in the world, in which visual elements move within causally connected reference frames. Friction renders velocities in these reference frames mostly stationary, formalized by an additional delta component (at zero) in the prior. Inverting this model automatically segments visual inputs into groups, groups into supergroups, progressively inferring structured reference frames and “perceives" motion in the appropriate reference frame. Critical model predictions are supported by two experiments, and fitting our model to the data allows us to infer the subjective set of reference frames used by individual observers. Our model provides a quantitative normative justification for key Gestalt principles providing inspiration for building better models of visual processing in general.

## Abstract (web summary)

"Motion perception depends on reference frames, but it is unclear what the brain’s reference frame is. Here, the authors show that human motion perception follows a hierarchical causal inference process, dynamically segmenting visual scenes into nested reference frames.”

## Introduction

If motion can only be defined relative to a reference frame^1^, what is the brain’s reference frame for the perception of a moving object? A century of psychophysical studies has provided us with evidence that motion is alternatively perceived in a retinotopic reference frame^2^, in allocentric (world) coordinates^3^, or coordinate frames defined by other objects in a visual scene^4–8^. Interestingly, perceived motion can rarely be explained by a single reference frame. For instance, in the famous Johansson illusion^4^, while the perceived velocity of the center dot is clearly biased away from the observed retinal velocity, it is not vertical as predicted by a reference frame defined by the flanker dots. Equally, the “flow-parsing” hypothesis^3^ proposes that the brain subtracts optic flow signals that are compatible with self-motion in order to make us perceive object motion in allocentric coordinates; yet, the empirically observed subtraction is rarely complete^9^.

Importantly, our perception of motion appears to be closely linked to an observer’s perception of the Gestalt of a scene, its structure, or configuration^10^. Previous work has made some progress in mathematically formalizing this elusive concept of a Gestalt: first in information-theoretic terms^5,11^, and more recently in closely related Bayesian terms^12–14^. A Bayesian formulation, compatible with the widely influential idea that the brain combines incoming sensory information with prior expectations to form subjective beliefs about the outside world^15,16^, has the advantage that these priors can be justified by the statistics and structure of the outside world. Yet, existing Bayesian accounts of motion perception were either formulated in a purely retinotopic reference frame^17,18^ or relied on a hierarchical prior that was inspired by the nested structure of the physical world but formalized as a two-parameter hierarchical Chinese restaurant process for computational convenience^7^, making it uncertain whether this formalization can adequately capture the complexity of real-world physics or be clearly interpreted in physical terms. Furthermore, quantitative empirical tests of some motion models have relied on explicit questions about structure identity (e.g., “Do you perceive structure A or B?”^7,19^). Other studies^20^ employ a motion prediction task in an experimenter-defined retinotopic reference frame which may differ from the reference frame underlying observer percepts. In both cases, these designs do not directly probe the velocity observers actually perceive, because they focus on either structure judgments or predictions within an experimenter-defined coordinate system rather than on the perceived motion itself.

On a mechanistic level, motion signals are processed locally in early visual areas, and the brain needs to combine information across these local motion detectors to form a coherent percept. A long line of research has modeled how these early areas detect local motion^2,21^ and how, through a series of linear-nonlinear stages, this local motion can be combined into a more global motion percept^2,22^. While integrating these local motion signals allows the brain to solve the ‘aperture’ problem^23,24^, it is not always useful for the brain to integrate information^25^. In fact, local motion differences are a powerful segmentation cue, and several studies^6,25^ have shown how our brain contrasts local motions to perceive relative motion. It is, however, unclear how the brain decides between these two opposing operations, integration and segmentation.

A separate line of research^26^ in multisensory integration has modeled how the brain solves a similar problem of deciding when to combine information across cues in a Bayesian framework (‘causal inference’). Given the general nature of this problem of deciding when to combine information, causal inference has been proposed to be a universal computational motif across the sensory cortex^27^. In motion perception, causal inference has been used to successfully explain biases in estimating heading from both visual and vestibular cues^28,29^, but it has not been applied to the perception of moving objects.

In this work, we present a hierarchical causal inference model that overcomes all of the above challenges by performing joint inference over the hierarchical structure of a scene and the motion of individual visual elements within it. Importantly, the motion priors in our model are justified by motion in the real world, in which most objects are not merely slow, but exactly stationary with respect to their canonical reference frame^16^. We also present data from two psychophysical experiments in humans that probe the hierarchical perception of motion and that provide strong support for the key elements of our model, in particular its hierarchical structure, proposed mixture prior, and approximate computations.

## Results

### Motion is perceived in dynamically inferred reference frames that reflect the causal structure of the world

**Fig. 1:**
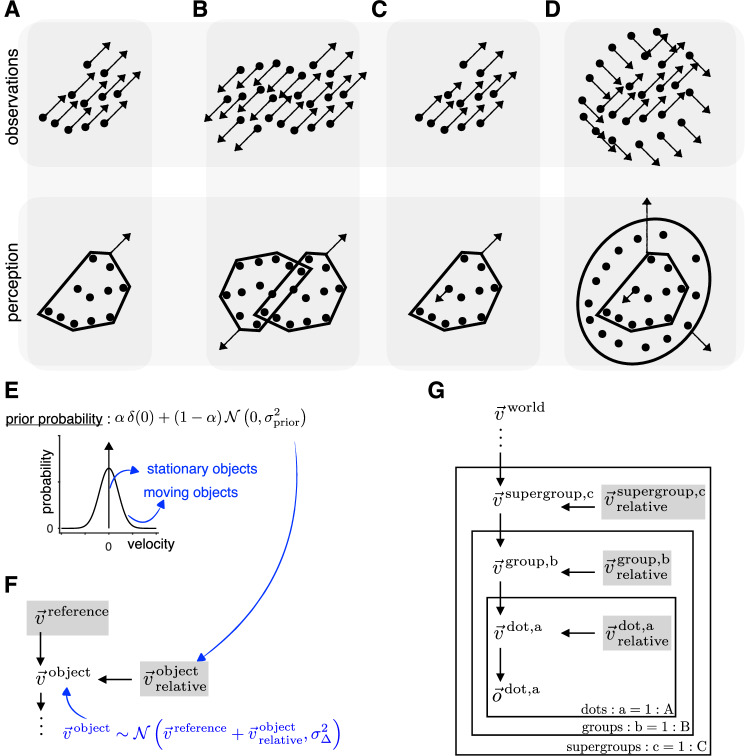
Causal inference model for hierarchical motion perception. **A**–**D** Illustration of four different dot patterns that are observed to move, as shown in the top row with the arrows indicating retinal velocity vectors. The bottom row shows our predicted motion percept, where the brain uses common velocity information to combine dots into objects and group objects into a hierarchical structure. **E** Prior that consists of a mixture of a delta distribution at 0 and a Gaussian distribution centered at zero, reflecting the knowledge that elements are either exactly stationary in an appropriate reference frame or are likely to move with a slow speed. **F** Generative model motif in which the object’s observed retinal velocity is the sum of the reference frame velocity (${\overrightarrow{v}}^{{{\rm{reference}}}}$) and its velocity with respect to the reference frame (${\overrightarrow{v}}_{{{\rm{relative}}}}^{{{\rm{object}}}}$). **G** Hierarchical causal inference model obtained by repeatedly applying the motif in (**F**). Inference in this model leads to the hierarchical grouping of dots, and representing dot motion in reference frames defined by the groups they belong to. The percept is determined by the non-zero relative velocity lowest in the hierarchy.

Much of the world consists of approximately rigid objects that, in turn, are made up of approximately rigid parts. During translation, all points on a rigid object move in the world at the same speed. Consequently, a common velocity for multiple moving elements in a visual scene acts as a strong cue that the elements belong to a single object. Not surprisingly, when we observe a group of dots moving at the same speed (Fig. 1A, top), our brain combines them into one object that it perceives as moving (Fig. 1A, bottom), rather than perceiving the individual parts as moving independently^10,30^. This common velocity cue also allows the brain to segment the scene into multiple moving objects. For example, when we observe the dots moving as shown in Fig. 1B, top, we perceive two partially overlapping objects moving at their respective velocities (Fig. 1B, bottom).

Importantly, objects do not simply move independently in the world but are related to each other through hierarchical whole-part relationships. When a part moves differently from the whole, the whole becomes a natural frame of reference in which to represent that part’s motion (Fig. 1C, D). For example, the body is the natural frame of reference for the motion of an arm because of the causal whole-part relationship between the two: any change in the motion of the body is directly translated into a change in the motion of the arm.

Our key idea linking retinal observations to percepts in Fig. 1A–D is that the brain dynamically constructs reference frames within which most of the visual elements it contains are stationary. We formalize this aspect of the physical world by extending the traditional slow-speed prior^18,31^ over moving objects to include a mixture component consisting of a delta at 0. This is shown graphically in Fig. 1E, where *α* denotes the prior probability that an object is stationary. This prior acts on the relative velocities of the visual elements represented in this reference frame. This brings us to the central motif in the generative model we propose is used by the brain to perform inference (Fig. 1F). The motif specifies how the velocity of an object is the sum of the velocity of the reference frame and the velocity of the object within that reference frame. This sum is probabilistic, allowing for computational imprecision as quantified by ${\sigma }_{\Delta }^{2}$. Inference within this generative model motif leads to a decomposition of the observed velocities of visual elements into the velocity of a (shared) reference frame and each element’s velocity relative to that reference frame. The degeneracy of this decomposition is broken by the mixture prior over the relative velocities, which leads to an automatic “chunking” of moving elements into groups that are inferred to move together. We hypothesize that the perceived velocity of a visual element moving in a reference frame is its relative velocity to the reference frame velocity if the relative velocity is non-zero and the reference frame velocity otherwise. This is compactly illustrated in our model by adding a shaded gray box around the candidate variables for perception in the generative model. Under this illustration, the percept corresponds to the candidate velocity that is non-zero and lowest in the model hierarchy (closest to the observations).

Recursively applying this motif leads to our proposed hierarchical causal inference model describing velocity percepts in a scene consisting of dots moving according to hierarchical causal relationships. Our model combines dots into groups, groups into supergroups, and so on (Fig. 1G). We define a grouping tree as a graphical tree in which nodes at each level are the groups and supergroups inferred by the model, and the edges show which dots form groups, which groups form supergroups, etc. The hierarchical causal structures relating the velocities in the scene are defined by specifying which nodes in the grouping trees are stationary (forming reference frames) and which nodes move in the appropriate reference frames (see Supplementary Methods B). At the bottom of the model are the actually observed velocities in retinal coordinates, $\vec{o}$, which are linked to the latent variables $\vec{v}$ by a Gaussian likelihood whose width represents the observational noise. The top level of the hierarchy is the velocity corresponding to the stationary objects in the world. The velocity of the stationary objects are perceived in the egocentric reference frame. For stationary observers, this is zero, but for moving observers, it is equal to the optic flow velocity caused by self-motion. This allows for a natural extension of this model to explain deviations in perceived velocities due to self-motion^3,32,33^.

Performing Bayesian inference in this generative model requires computing a posterior belief over all possible causal structures in which the visual elements could be grouped, and over all the velocities in each of the structures. Before empirically testing the quantitative predictions of this model, we next explain the intuitive impact of its key elements using an increasing number of moving dots, building up to explain the classic Johansson illusion (Supplementary Video 1).

### Illustrating the causal inference model for stimuli consisting of 1-3 dots

**Fig. 2:**
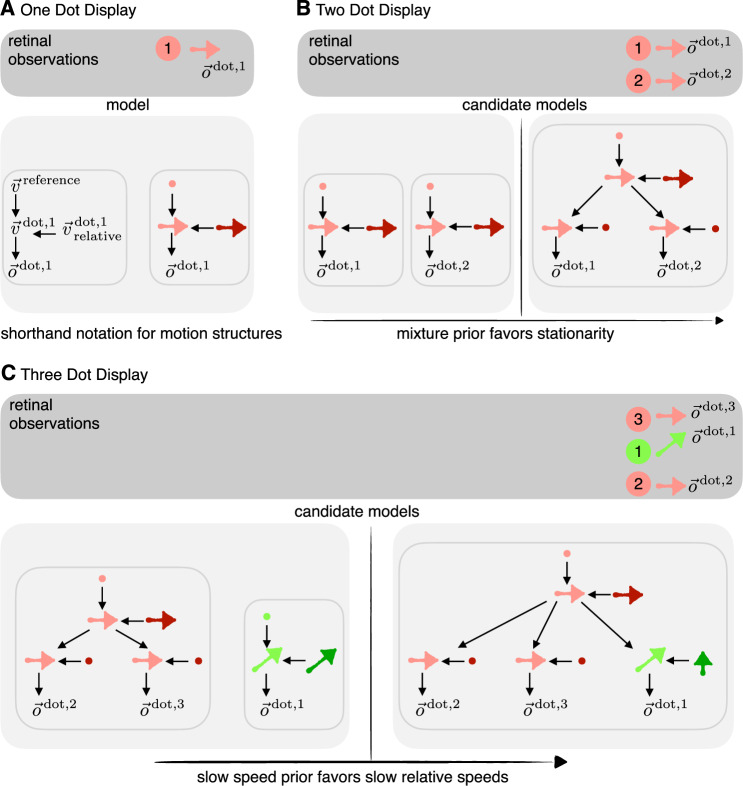
Model predictions for simple dot stimuli. **A** The motion of a single dot is inferred in the reference frame of a stationary world. In our shorthand notation, velocity variables have been replaced by their most likely value shown as a motion vector. Darker shades are used to indicate relative velocities, and filled circles indicate zero velocity. **B** Two moving dots are explainable by two possible structures (left and right). If they move coherently, such that both are stationary with respect to a moving group, the delta component in the prior implies that most posterior mass lies on the combined structure (right). As a result, we perceive a moving object consisting of both dots. **C** If a third dot is added to the display in (B), the observations are explainable by 264 different structures (Methods, and Supplementary Methods B), two of which are shown here. On the left, the green dot is perceived as independent of, and unaffected by the motion of the red dots. On the right, the green dot is part of the same structure as the red dots and perceived in a reference frame defined by a group in which two out of the three dots are stationary (favored by the delta component in the prior). The Gaussian component of the prior favors the right structure over the left one since the velocity of the green dot in the reference frame defined by the red dots is smaller than its velocity with respect to the stationary world. This explains the Johansson illusion^4^.

We illustrate the key features of the model by applying it to very simple stimuli consisting of two or three dots. The model infers full posteriors over all possible structures and the velocities within each structure. For compactness, we focus on the most probable structures and show the most likely inferred velocity using vectors instead of variables in the generative model, as shown in Fig. 2A for a simple stimulus in which a single dot is moving. We explicitly show the variables for the rest of the structures in Supplementary Fig. S1.

When observing two dots that move with the same velocity, there are two likely structures that can explain the observations: one in which both dots are moving independently, each represented in the egocentric coordinate system as an individual dot (Fig. 2B, left structure), or a structure in which each dot can be inferred to be stationary with respect to a group (an abstract object), consisting of both dots, with the velocity of the group corresponding to the retinal velocity of the dots (Fig. 2B, right structure). The delta component of our mixture prior over the relative velocities ensures that the latter hierarchical structure has the highest likelihood given the data since it has the fewest non-zero relative velocities. Furthermore, since the observed velocities for each dot will slightly deviate from each other due to observation noise, the group velocity combines both observations to obtain a more reliable velocity percept of the group^34^. This combination of dots into a single group occurs for all dots in the scene that are inferred to move with the same velocity.

If a third dot is added to the scene that moves with a different velocity from the other two dots, the two likely structures to explain the observations are: (a) an object consisting of the two coherently moving dots plus an independently moving third dot (Fig. 2C, left) and (b) a single object consisting of all three dots in which the differently moving dot is represented as moving in the object’s reference frame (Figure 2C, right). The slow speed component in our mixture prior favors the latter structure if the differently moving dot has a smaller speed in the object’s reference frame than in the stationary egocentric reference frame. For instance, in the Johansson illusion (Fig. 2C), the third dot has a small relative velocity with respect to the two coherently moving dots, and its perceived velocity is indeed biased towards its velocity in the reference frame provided by the group made up of the two dots (dark green vertical arrow in Fig. 2C, right). However, it is important to recognize that even for as little as three moving elements, there are 264 different causal structures, for instance, one in which dots 2 & 3 move relative to dot 1 rather than the other way around, or where all dots move relative to a reference frame defined by all of the dots together, moving at an intermediate velocity (for more information, and a derivation of the number of causal structures for *n* moving elements, see Supplementary Methods B). So it may not be surprising that structure (b) alone cannot explain human observers’ percepts which sometimes correspond to the retinal velocity suggesting structure (a), sometimes correspond to the relative velocity (structure (b)), and sometimes lie in between^35,36^, suggesting that the brain performs inference over multiple, if not all, of these possible structures.

Furthermore, model predictions will depend on how perception is related to the posterior over structures and velocities. Prior work^7^ has suggested the mean for the most likely structure, but it could also correspond to the mean across structures as in other work on causal inference^26^, or posterior sampling^37,38^.

### Empirical tests of model predictions

In order to quantitatively distinguish between our model and previous proposals, we designed two motion estimation tasks that tested the key elements of our model: (i) the proposed mixture prior over relative velocity with a delta at zero, and (ii) the linking hypothesis mapping the posterior distributions over velocities in the model to the distribution over perceived velocities. Importantly, by using a motion estimation task, we test both causal inference over reference frames and the perception of motion within a reference frame.

#### Experiment 1: Test using stimuli with two potential levels of hierarchy

**Fig. 3:**
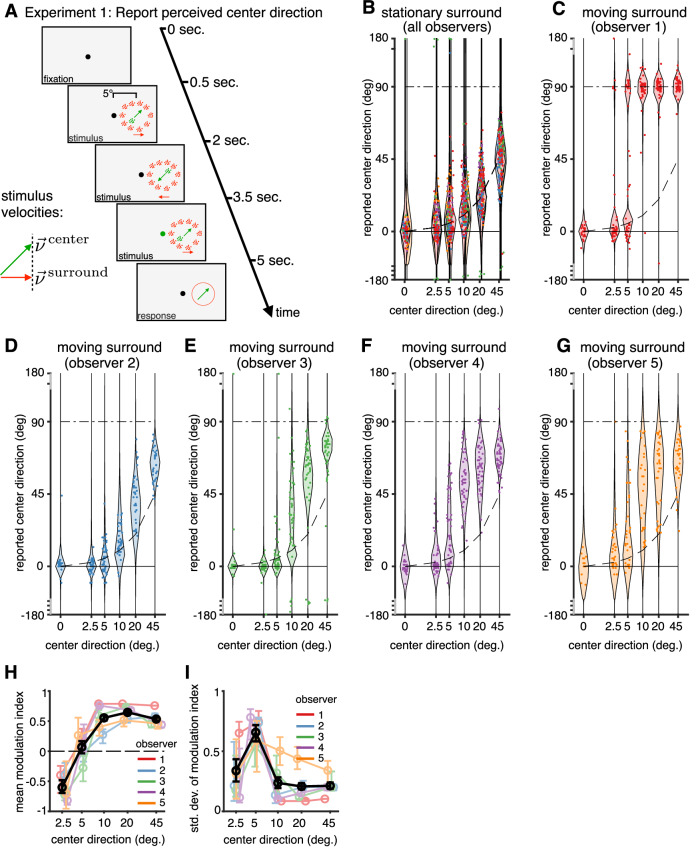
Experiment 1 – design and results. **A** During fixation, two groups of dots (red and green) appear and move back and forth three times over a 4.5 second viewing period before disappearing. During the last phase of movement, the fixation dot turned green. The observers adjusted a dial to report their perceived direction of the green dots during the last movement phase. The red (surround) dots were either stationary or moved horizontally (0^∘^), while the green (center) dots varied in direction from trial to trial while keeping the horizontal component of their velocity matched with the surround. **B** Responses of all five observers overlaid for the condition where the surround is stationary. Each dot represents a single trial. All responses lie around the dashed identity line (warped due to non-linear spacing of the *x* − axis). Responses were flipped for negative center directions to match the positive directions after verifying that the responses were symmetric. **C**–**G** Responses for each observer when the surround is moving. The horizontal lines at 0^∘^ and 90^∘^ indicate the predicted reports for complete integration (perceiving the surround) and complete segmentation, i.e., perceiving the relative velocity, respectively. **B**–**G** The overlaid violin plots show the model predictions (not data distributions). One model was fit jointly to all data for each observer. **H**, **I** Mean (H) and standard deviation (I) of the modulation index (median + 68% confidence intervals, with an average of *n* = 66 data points going into the computation for each center direction) defined such that  − 1 corresponds to pure integration,  + 1 to pure segmentation, and 0 to retinal motion. Different colors indicate different observers; the black line denotes the average across observers.

In order to test our model, and to constrain its parameters, Experiment 1 was designed to precisely measure human motion perception during the transition from integration to segmentation. Observers used a dial to report their perceived motion direction of a patch of green dots surrounded by a variable number of patches of red dots (Fig. 3A). Surround dots were either stationary or moving horizontally (0 or 180 degrees). The number of surround patches was randomly chosen every trial from {1, 2, 3, 5, 10}, while the center always consisted of a single patch of dots. In addition, the retinal direction of the center patch (direction on the screen) was randomly chosen on each trial from the set {0, ± 2.5, ± 5, ± 10, ± 20, ± 45}°. The center and surround had a common horizontal velocity (0° or 180°), such that the direction of the center’s velocity relative to the surround was  ±90° depending on the sign of the center direction (more details in Methods). As expected, reported directions lie along the identity line when the surround is stationary (Fig. 3B).

When the surround is moving, observer responses systematically deviate from the identity line (Fig. 3C–G and Supplementary Fig. S4). Specifically, responses appear biased towards zero degrees (surround direction) for small center directions for most observers, consistent with the observer integrating the center and surround velocities and reporting the cue-combined velocity. The reported velocities are biased towards 90° for larger center directions, consistent with the observer perceiving the relative velocity between the center and the surroundings.

We quantify the effect of surround motion on reports about center motion by mapping the responses to a modulation index. This index lies between  − 1 and  + 1, where  − 1 corresponds to complete integration (perceiving the surround direction), 0 corresponds to the surround having no effect, and  + 1 corresponds to complete segmentation (perceiving the relative velocity between the center and surround; see Methods for details). The mean modulation index, averaged across observers (Fig. 3H), is negative for a center direction of 2.5°, indicating integration (*p* < 0.001 for the group, with *p* < 0.05 individually for 4 out of 5 observers, i.e., *p* < 0.001, *p* < 0.001, *p* = 0.006 and *p* = 0.048, based on 5000 bootstrapped samples). For larger separations (greater than 10°), the average and individual mean modulation indices are positive, indicating segmentation for larger separations (*p* < 0.001, based on 5000 bootstrapped samples). The standard deviation of modulation index, averaged across observers (Fig. 3F) is largest for intermediate separations (standard deviation at 5° is larger than 2.5° and 45° (*p* = 0.012; based on 5000 bootstrapped samples) indicating a higher variability due to uncertainty over causal structures, in agreement with our model predictions.

Finally, we fit our model to the data by inferring posterior distributions over the model parameters given the data from each observer. We find that the empirical responses are in excellent agreement with the response distributions predicted by our causal inference model, which are overlaid as violin plots in Fig. 3C–G. The goodness of fit was measured by variance explained (VE) to be between 92–96% across observers. Observers’ showed increased variability (bimodal responses for some) for intermediate center directions in both empirical responses and model fits. This is consistent with predictions of the causal inference model, which infers greater uncertainty about whether to integrate or segment for intermediate center directions. These characteristic deviations between retinal motion and perceptual reports depend on the number of surround patches, also in agreement with model fits (Supplementary Fig. S3 with an average VE of 94%).

#### Insights from model fitting

Fitting the model to data allowed us to gain three key insights about the model: (a) whether the mass in the delta component was required to explain the pattern of responses, (b) how observers map the inferred posteriors to responses on each trial, and, (c) how the different causal structures contribute depending on center direction.

**Fig. 4:**
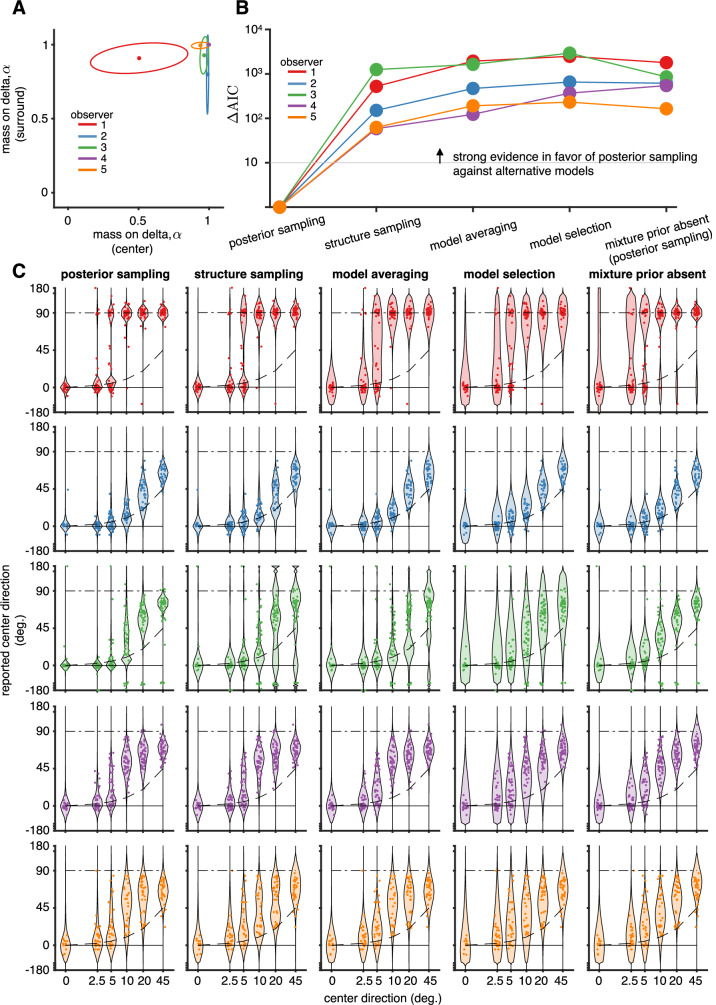
Model fitting insights from experiment 1. **A** Fitted mass on the delta component in the prior for center and surround. Each colored line shows the mean and 95% CI for each observer evaluated using an average of *n* = 1446 data points for the computation of the posterior. **B** Model comparison. For each model, the difference in AIC score to the posterior sampling model is shown. We find strong evidence against all alternative models as compared to the posterior sampling model. **C** Model predictions overlaid with data for each model considered in (**B**).

Remarkably, for all observers, most of the prior mass was in the delta component, indicating the strength of the brain’s expectation that relative motion in the world is exactly zero, rather than merely slow (Fig. 4A, *α* close to 1). We further confirmed the presence of a delta component in the mixture prior by a formal model comparison (Fig. 4B, ‘mixture prior absent’ model) and found strong evidence against a model without mass in the delta component.

Our model comparison (Fig. 4B) also revealed that observers’ responses are best described as arising from approximate inference (posterior sampling) in the full Bayesian model. We compared this to other previously proposed mappings from posteriors to responses: (a) reporting the posterior mean (model averaging) which is the optimal strategy for estimation tasks with L2-loss^39^, (b) reporting the conditional mean under the most probable structure (model selection) which maximizes consistency^40^ and minimizes the description length^41^, and (c) reporting the mean by sampling the structure (structure sampling) which is a more precise approximation than posterior sampling^38^. Among these models, previous studies on hierarchical motion perception^7,42^ have mapped posteriors to reports using the model selection strategy. We also tested whether the proposed delta component at zero in the prior over velocities was actually needed to explain the data by comparing each of the models with and without the delta in the prior in a comprehensive, factorial model comparison (Supplementary Fig. S8). In Fig. 4, we only show the results for five models: four models that include the delta at zero, one model for each mapping from posterior to perceptual report, and one model that omits the delta at zero and uses posterior sampling.

The predicted response for each of these models is shown in Fig. 4C, overlaid with observer responses. Structure sampling reports the mean under the sampled structure. Thus, any variability conditioned on the structure must be due to observation and motor noise which are shared across all structures (and cannot be ascribed to variability due to sampling conditioned on the structure, as for posterior sampling). This leads to larger-than-required predicted variability for some center directions (seen prominently in observer 3), reducing the likelihood of the data under this model. Model averaging, which reports the weighted mean across structures, increases this problem since averaging reduces variability. Furthermore, it also predicts intermediate reports that largely fail to explain bimodal features in the data (seen prominently in observer 1). Model selection reports the posterior mean within the structure with the highest posterior probability. While able to explain bimodal reports due to observation noise varying which structure is most likely, it overestimates observation and motor noise for the same reason that structure sampling and model averaging do: the low variability of the mean velocity conditioned on the structure (seen prominently in observer 1). Due to the absence of the delta prior in the mixture-prior-absent hypothesis, the predicted responses show weaker segmentation, and increased observation noise in order to capture the increased variability in the data during the transition (the model fit for observer 1 tried to attribute the responses around 0 to a constant lapse, hence showing some bimodal signatures).

#### Inferred structures in experiment 1

**Fig. 5:**
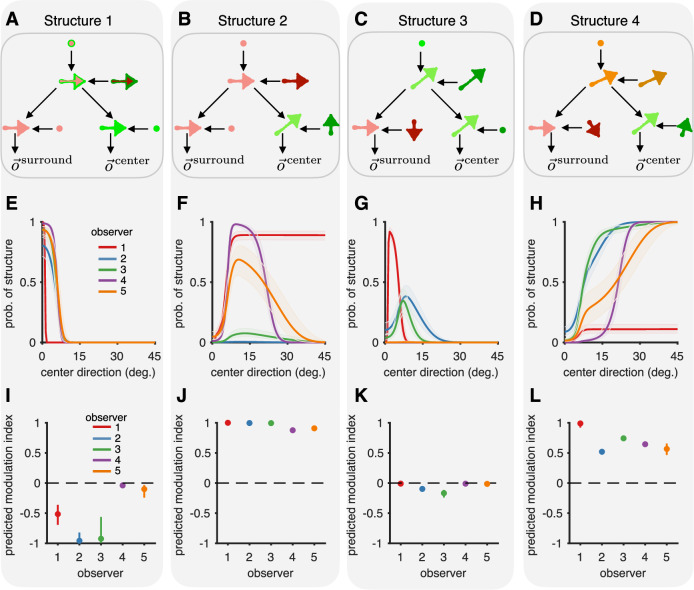
Inferred structures for observers in experiment 1. **A**–**D** The four causal structures having significant posterior mass in our model fits. See main text for description. **E**–**H** The posterior probability (median along with 95% CI evaluated with an average of *n* = 1446 data points going into the computation of the posterior) assigned to each structure by each of the observers, as a function of center direction. All observers integrate the center and surround for center directions close to zero (A + E) and segment the center and surround otherwise. However, they differ in the degree to which they rely on each of the three different reference frames implied by (**B**, **C**, and **D**). **I**–**L** As in Fig. 3H, the modulation index (median along with 95% CI evaluated with an average of *n* = 1446 data points going into the computation of the posterior) predicted under each structure quantifies the influence of the surround on the perceived center direction. The modulation index for a structure is independent of the probability assigned to that structure. Together, they determine the influence of the surround on the center.

Our model also allowed us to determine the structures underlying each observer’s subjective percepts. We found that, for all observers, only four out of 12 possible structures (Supplementary Fig. S2) were assigned a significant posterior mass (Fig. 5A-D for 10 surround patches, and Supplementary Figs. S6, S7 for all other conditions). Under structure 1, the observer integrates center and surround, thus perceiving the cue-combined velocity (Fig. 5A). As expected, the posterior probability of this structure was highest when the center and surround moved with the same velocity and decreased with an increase in separation between center and surround velocities (Fig. 5E). The bias in the perceived center velocity towards the surround was determined by the weight given to the surround (Fig. 5I), which we quantified using the modulation index (as in the previous section). Under Structure 2, center motion is perceived in the reference frame defined by the surround – the canonical structure typically assumed for center-surround motion segmentation (Fig. 5B, F, J). Unexpectedly, this structure is dominant for only 1 out of 5 observers, plays a transient role for intermediate differences between center and surround motion directions for just 2 observers, and only plays a very minor role for the remaining observers. The same is true for structure 3, under which the surround is perceived in the reference frame of the center, and perception of center motion mostly coincides with retinal motion (Fig. 5C, G, K). Finally, structure 4 implies that both center and surround motion are perceived with respect to a reference that moves at a velocity intermediate to both center and surround (Fig. 5D, H, L). This is the structure that carries primary responsibility for intermediate percepts (i.e., the apparently incomplete subtraction of the surround from center velocity) at large differences between center and surround motion directions. Surprisingly, none of the observers places any mass on the possibility that the center and surround might belong to different causal structures (the 99th percentile of mass on this structure is below 1% for all observers) – an alternative potential explanation for intermediate percepts. However, one prediction of such a structure would have been to find a substantial fraction of responses along the identity line (given that responses are best explained by posterior matching overall), something that we did not observe (Fig. 3C–G). The reason that structure 4 has higher posterior mass than structures 2 and 3 for large separations is the Gaussian slow speed component in the prior: the smaller relative velocities understructure 4 overpower the mass in the delta component of the prior.

As predicted by the model, we also find that both the integration effect (quantified by the model predicted modulation index at 0°) and segmentation effect (quantified by the model predicted modulation index at 45°) become stronger with increase in the number of surround patches (Supplementary Fig. S9B and S9C respectively). We also find that the transition from integration to segmentation shifts to larger separations with increase in the number of surrounding patches (Supplementary Fig. S9E).

#### Experiment 2: Test using stimuli with three potential levels of hierarchy

Next, we added a second surround of moving dots to test three key qualitative behavioral predictions of the hierarchical replication of our causal inference motif (different grouping trees shown in Supplementary Fig. S10). First, motion perception during segmentation is local, i.e., the perceived motion of an element inferred to be moving relative to a group is independent of other motion in the visual scene. Second, if a visual element is integrated with a group, then it inherits the reference frame of that group. Third, the retinal velocity of a group, and not the velocity with which the group is perceived, determines the reference frame velocity for the elements that are part of that group (compare the alternative model in Supplementary Fig. S18 and Fig. 1G).

**Fig. 6:**
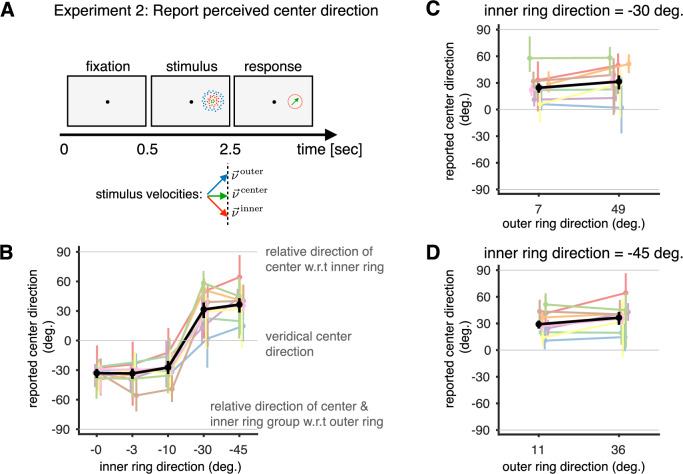
Design and results of Experiment 2 (three moving groups). **A** The observer performs an estimation task in which they have to report the direction of green dots (center) using a dial. While center dot directions are randomized from 0 to 360^∘^, results are combined after rotating all velocities such that ${\overrightarrow{v}}^{{{\rm{center}}}}$ moves horizontally (0^∘^). **B** Our model predicts that we will perceive the center dots in the inner ring’s reference frame if both move in noticeably different directions (segmentation, gray line at 90^∘^) and cue combine the center and the inner ring motion if they are sufficiently similar (integration, gray line at  − 90^∘^). In the latter case, our percept would be the cue-combined center and inner ring’s velocity in the outer ring’s reference frame. The observer responses (circular marginal median plotted with 95% CI, with an average of *n* = 41 data points used in the computation for each inner ring direction) support these model predictions (the black line shows average response across participants). **C** The causal inference model predicts that during segmentation, the percept of center motion should only depend on the retinal inner ring motion, not the perceived inner ring motion. The data (circular marginal median plotted with an average of *n* = 41 data points used in the computation for each outer ring direction) clearly supports this prediction since the outer ring motion has no influence on the reported center directions (even though it influences the inner ring percepts, see Supplementary Fig. S19). **D** Same as (**C**) but for conditions where the inner ring moves at  − 45^∘^ and the outer ring moves at either 11^∘^ or 36^∘^. We consider the two inner velocities that are most different from the center in our experiment to minimize the probability that the observer integrates the center and the inner ring velocities.

As in Experiment 1, observers reported the direction of the center patch moving in the presence of a surround; however, in Experiment 2, the surround consisted of inner and outer rings of dots (Fig. 6A). The inner and outer ring velocities were on opposite sides of the center patch velocity with the inner ring moving at {0°, − 3°, − 10°, − 30°, − 45°} clockwise from the center. The outer ring moved at 60° counter-clockwise from the center when the inner ring moved at 0°, and the outer ring’s velocity was adjusted to maintain a constant relative velocity between the outer and inner rings as the direction of the inner ring was varied. This design was motivated by the goal to find a clearer empirical signature of the integration process than possible in Experiment 1. When the center and inner ring are integrated, we expect them to be perceived relative to the outer ring, leading to perceptual reports that are biased towards  − 90°. However, if the center and inner ring and perceived to move independently, the responses would lie around 0°. When the center is segmented from, and perceived relative to the inner ring, we expect perceptual reports biased towards  + 90°, just as in Experiment 1.

Since there are many more possible causal structures that could explain our stimulus, we would ideally make quantitative predictions using our full model. However, we found this intractable due to the large number of possible causal structures (264, see Supplementary Methods E). Thus, we generated qualitative predictions (Supplementary Fig. S11) by making two simplifications: First, we only included structures in which every group had at least one stationary member (‘pure’ structures, Figs. S12–S17) – all the remaining structures predict percepts in between the percepts of these pure structures. Second, we assumed that the uncertainty about the inner ring velocity was much lower than that for the center and that the uncertainty about the outer ring velocity was much lower than that about the inner ring – justified by the 5 fold increase in number of dots from center to inner ring, and inner ring to outer ring.

The data clearly confirmed our expectations and qualitative model predictions: when the center and inner ring are integrated to form a group, this group is perceived in the reference frame formed with the outer ring (Fig. 6B). In our stimulus design, the velocity of the group formed by the center and inner ring is always clockwise to the center ring velocity (with  − 90° the relative direction in the outer ring’s reference frame). We see clear signatures of integration of the center and inner ring into a group (Fig. 6B) across participants (*p* < 0.001 based on 10^4^ bootstrap samples), with *p* < 0.01 individually for all observers (with max *p* = 0.002 based on 10^4^ bootstrap samples) for smaller separations (≤ 3°). For larger separations between the center and inner ring, the model predicts that the center is perceived as moving in the reference frame formed with the inner ring, ignoring the outer ring. In our stimulus, this translates to reports being biased counter-clockwise to the center ring, which we observe across participants (*p* < 0.001 based on 10^4^ bootstrap samples), with *p* < 0.01 individually for 7 out of 9 observers, (with max *p* = 0.003 based on 10^4^ bootstrap samples).

Our data also support our model’s prediction that the retinal velocity of the inferred group determines the group’s reference frame velocity (Fig. 6C and D, respectively). Specifically, as the center is perceived relative to the inner ring when they move differently, the outer ring should have no effect on the perception. Consistent with this, we found no significant difference in reported center directions for different outer ring directions, for inner ring directions of  − 30° and  − 45° (*p* = 0.23 and *p* = 0.16 respectively, evaluated through bootstrapping, using 10^4^ resamples, with only 1/9 observers showing significant difference for both inner ring directions with *p* = 0.03). For these inner ring directions, the observers predominantly segment the center and inner rings (Fig. 6B). The individual trial responses are shown in Supplementary Fig. S19.

In addition to the conditions shown in Fig. 6B, we also test how: (a) observers perceive the inner ring in the presence of the center and/or surround, (b) how the center ring percept is biased by just the inner ring moving coherently (similar to Experiment 1) and just the outer ring moving coherently. Responses in these conditions also agree with predictions of the causal inference model (Supplementary Fig. S20C–J).

## Discussion

We have presented a model of complex motion perception, and new data to support this model. The key normative ingredients, reflecting the structure of the physical world, are that the motion of visual elements should be represented in reference frames that they are causally connected to, that due to friction forces, the velocity in such a reference frame is mostly zero, and that the world is compositional and hierarchically organized. We designed two experiments in order to test all key elements of our model, to constrain its parameters, and to generate insights for motion perception in scenes with rich motion structures.

Our work advances the large body of work trying to understand sensory processing in terms of natural input statistics^43–46^. Whereas prior work started with the input statistics at the sensory periphery, our model explicitly incorporates causal relationships (moving the whole will move the part) and the importance of friction (the relative velocity is zero for most objects that are touching each other) for giving rise to these statistics. Strikingly, fitting our model to perceptual reports revealed that most prior mass is in fact, at zero for all of our human observers (Fig. 4A).

Our work was strongly inspired by ref. ^7^ who embedded the vector decomposition idea of ref. ^4^ in a Bayesian inference framework and extended it both hierarchically and spatially. We built upon their model in three ways: first, we changed the structure of the model so that inference only required local computations. Second, this allowed us to define prior distributions over the relative velocities, and to use known physics to inform them by including a delta-component at zero. This prior over velocities also implied a prior over causal structures that differed from: (a) the Chinese Restaurant Process previously assumed by^7^ that placed elements in a group in proportion to its size, and (b) from related works^20,42^ that used a Jeffrey’s prior over motion strength to penalize the levels of hierarchy^20,42^. Finally, we allowed for a flexible mapping from posteriors over structures and velocities to perceptual reports. Our data strongly constrained this mapping to correspond to sampling over both structures and velocities within each structure, different from the MAP inference over structures assumed in related prior work^7,19,20,42^.

The richness of our psychophysical data, together with formal model comparison, also allowed us to answer the important question of how the often-complex posterior distributions of a Bayesian observer are related to our deterministic-appearing percept^47,48^. In prior work, percepts were modeled as averages over velocity within the most probable structure only (‘model selection’, i.e., MAP inference over structures)^7,42^. Our experiments and analyses provide overwhelming evidence that, at least in the context of motion processing, the brain performs joint inference over both structure and motion and that perceptual reports are best described by samples from this joint distribution (Fig. 4B).

Approximate joint inference in our model can also explain why effect sizes in prior studies usually deviated from previously proposed theories like (Bayesian) Vector Analysis^4,36^ or flow-parsing^3^: a range of different causal explanations are all consistent with the presented impoverished psychophysical stimuli. We confirmed a closely related prediction of our model – the increase in integration and segmentation effect sizes with the number of dots, i.e., the uncertainty in the surround^49^ of our experiment 1 (Supplementary Fig. S9).

The mixture prior over velocity that we propose extends the slow-speed prior previously proposed by^17^ and quantitatively investigated by^18^. Strikingly, we found that most of the prior mass was in the delta component not included in earlier work. Most likely, the delta component was not required to explain the data in ref. ^18^ since the speeds used in their experiments were too large for the delta component to matter. On the other hand, their data suggested a prior shape of speeds with heavier tails than the prior used by us (our Gaussian prior over velocity implies a Raleigh distribution over speed). Future work should combine a delta at zero with a heavier-tailed distribution over velocity to better match data at large velocities.

Fitting our model to individual responses revealed unexpected variability in the causal structures inferred by different observers. We had expected that partial segmentation for large differences in motion direction between the center and surround was best explained by observers’ lack of confidence that the center and surround were indeed part of the same object. However, our quantitative analysis showed this not to be true: partial segmentation was actually explained by observers perceiving both center and surround to be slowly moving with respect to an abstract reference frame moving with an intermediate velocity. This result demonstrated the importance of the ‘slow-speed’ component of the prior on relative motion – in contrast to the otherwise analogous spike and slab (flat) prior^50^ common in machine learning.

In our stimuli, center dots had a different color from the surround in order to minimize observer confusion about which motion direction to report. This difference in color might have increased the probability of segmenting the center from the surround (we found anecdotal evidence during pilot sessions that perceiving and reporting the center motion was difficult when the surround had the same color). Using our experimental paradigm and model-based analysis, the effect of color, as well as the effect of other cues like spatial relationships, disparity, etc., can be quantitatively studied and related to model parameters like the prior probability of inferring the same motion, or the same causal structure. Furthermore, since our work was entirely based on the perception of motion directions, future work should also test our model predictions for speed perception. We also presented our stimuli in the periphery to prevent observers from tracking the dots, resulting in previously studied biases^51^, and to facilitate future neurophysiological studies in areas like MT. Future work can study the empirical influence of eye movements and incorporate them in our model as self-motion signals at the top level (currently assumed to be stationary).

While it has long been recognized that both integration and segmentation are key operations underlying motion perception^25^, our model shows that there are in fact, two qualitatively different kinds of segmentation: (1) do two visual elements belong to the same causal structure, and, if the answer is yes, (2) is a visual element moving with respect to its reference frame? While our model answers both questions using the framework of ‘causal inference’ from multisensory integration^26^, which has been proposed as a ‘universal computation’ for cortex^27^, only the first question corresponds to a question about causality as statistically defined^52^.

Our model also lends itself to making predictions for neurophysiological data. We leave for future work the tantalizing possibility that the two kinds of variables in our model (corresponding to the left and right sides of Fig. 1G) are represented by two major classes of neurons that have been described^53^ in cortical motion area MT (surround-suppressed and not surround-suppressed).

Bayesian causal inference has recently been proposed as a unifying theory for neuroscience^27^. Our model extends the simple ‘same’ or ’different’ scenarios in previous causal inference work to hierarchical whole-part relationships reflecting the compositionality of the world^54^. The computations at the lowest level of our model, in fact, resemble a recent probabilistic model of neural responses in the primary visual cortex^55^, and the hierarchical architecture of our model directly suggests an equivalent one for the ventral stream, potentially allowing us to understand both dorsal and ventral stream as performing inference in closely related generative models.

## Methods

### Observers

Five naive observers participated in Experiment 1, and 10 naive observers participated in Experiment 2. Observers provided written informed consent and were financially compensated for their time. Experiments were approved by the Office for Human Subject Protection (OHSP) at the University of Rochester (IRB number 0003909). 1/10 observers in Experiment 2 was excluded based on their large response variability (standard deviation greater than 30°) in the control condition, and their data was omitted from further analysis.

### Experiment 1 details: two moving elements

The stimulus consisted of a ‘center patch’ of green dots, presented at 5-degree eccentricity. Dots were 0.1 degrees in diameter and distributed uniformly within the patch with a density of 6.88 dots/degree^2^. The center patch had a radius of 0.68 degrees and was surrounded by a ring of radius 2.72 degrees, consisting of a variable number of patches of red dots (Fig. 3A). Each surround patch had a radius of 0.54 degrees and a dot density of 10.91 dots/degree^2^. Dot displays were viewed binocularly, and no disparity cues were added, such that all of the dots moved in the plane of the display. The stimuli were presented on a 27-inch monitor with a refresh rate of 60 Hz and a resolution of 1920 × 1080 at a viewing distance of 105 cm. Eye movements were tracked using an Eyelink 1000 system, and trials were discarded if the eyes moved within 1 degree of the center patch.

The stimuli were presented at an eccentricity of 5 degrees in the periphery. The number of dots in a patch was fixed to 10. There was one center patch and the number of surround patches was chosen in every trial from the set [1,2,3,5,10]. The center retinal direction was chosen from the set [0°, ± 2. 5°, ± 5°, ± 10°, ± 20°, ± 45°], and the surround was either stationary (stationary surround always had 5 patches) or moved at 0 degrees (horizontally rightwards) at a speed of 1 degree/sec. This relatively slow speed was chosen to maximize the effect size during initial piloting and is consistent with earlier studies that found stronger motion repulsion effects at slower speeds^56^. This speed dependence of effect magnitude is predicted by our model: center and surround become less likely to be causally connected at higher speeds due to the higher relative velocity.

After a fixation period of 0.5s, the stimulus appeared and moved back and forth for 1.5 cycles. The patch envelopes moved at a constant velocity and reversed their velocity after 1.5 seconds (square wave velocity profile with a time period of 3 seconds). The back-and-forth movement ensured that the envelopes stayed within a fixed area of the screen. The back and forth movement was also chosen to increase the observer’s belief that the center and surrounding dots were causally linked. In the last half cycle, the fixation dot turned green, indicating that the observer had to report the direction during the last half cycle. The key conditions are shown in Supplementary Videos 2-4.

After stimulus offset, an arrow appeared at the location of the center patch, and observers used a Griffin NA16029 dial to adjust the arrow direction to match their motion direction percept. The stationary surround condition served as a control. Observers who had a response standard deviation larger than 30 degrees in this condition were removed from subsequent analysis. The fixation period and stimulus had a total duration of 5 seconds following which the observers could make a response at any time to proceed to the next trial. Each observer participated in three sessions to get 22 trials per condition on average, for a total of 1446 trials per observer on average.

### Experiment 2 details: three moving elements

In experiment 2, the stimulus consisted of a center patch, an inner ring, and an outer ring, separated by 0.5 degrees, that were arranged concentrically and centered at 5 degrees eccentricity (Fig. 6A). On every trial, either the center dots or the inner ring dots, were colored green, indicating that their direction was to be reported. The other parts of the stimulus contained red and blue dots, respectively, with the color assignment randomly drawn every trial. The number of dots in the center was 10 (density of 3.2 dots/degrees^2^ with a center patch radius of 1 degree), the inner ring contained 50 dots (density of 4 dots/degrees^2^ with an inner ring width of 1 degree), and the outer ring contained 250 dots (density of 11.4 dots/degrees^2^ with an outer ring width of 1 degree). The viewing distance, eye recording details, and the criteria for discarding trials due to fixation breaks and control condition response variability were the same as in experiment 1.

Each trial started with a 0.5 s fixation period during which only the fixation dot was shown on the screen. This was followed by the random-dot stimulus, which moved for 2 s within an aperture at a constant velocity. Unlike for Experiment 1, the moving dots were presented inside fixed apertures, and the stimulus direction was not reversed. The center moved with a speed of 0.5 degrees/sec^56^, and the center direction was randomized across trials, within a range of 360°, to minimize the effect of reporting biases. The inner ring’s retinal direction was chosen from the set [0°, − 3°, − 10°, − 30°, − 45°] relative to the center, where negative angles indicate clockwise rotations. The outer ring moved in a counter-clockwise direction chosen such that the same relative velocity was maintained between the outer and inner rings across different inner ring directions. Furthermore, the speeds of the inner and outer rings were chosen such that the relative velocity between either ring and center was perpendicular to the center direction. The key conditions are shown in Supplementary Videos 5–9.

Different conditions were interleaved across trials in which the inner and outer rings could either move randomly or coherently, and the observer had to report the direction of the center or inner ring. As in experiment 1, the observer made their responses by adjusting the direction of the arrow that appeared at the location of the center or inner ring after the stimulus offset with its size matched to the size of the corresponding target. Each observer performed three sessions to get 46 trials per condition on average for a total of 2239 trials per observer on average.

### Modulation index to summarize observer responses in Experiment 1

We defined the modulation index, *w*_MI_, such that the percept of the center patch (${\vec{v}}_{{{\rm{percept}}}}^{\,\,{{\rm{center}}}}$) predicted for a given *w*_MI_ is given by

1 $${\vec{v}}_{\,{{\rm{percept}}}}^{\,{{\rm{center}}}}=\left\{\begin{array}{ll}{\vec{o}}^{\,{{\rm{center}}}}-{w}_{{{\rm{MI}}}}{\vec{o}}^{\,{{\rm{surround}}}}\hfill \quad &{w}_{{{\rm{MI}}}}\ge 0\\ (1+{w}_{{{\rm{MI}}}}){\vec{o}}^{\,{{\rm{center}}}}-{w}_{{{\rm{MI}}}}{\vec{o}}^{\,{{\rm{surround}}}}\quad &{w}_{{{\rm{MI}}}}\le 0\end{array}\right.$$

where ${\vec{o}}^{\,{{\rm{center}}}}$ and ${\vec{o}}^{\,{{\rm{surround}}}}$ are the observed center and surround velocities from the brain’s perspective for a given trial. This definition incorporates partial subtraction of the surround (case *w*_MI_ ≥ 0) for relative velocities when the effect on perception is repulsive^57^ and cue combination^58^ when the effect is attractive (case *w*_MI_ ≤ 0). Under the simplifying assumption that ${\vec{o}}^{\,{{\rm{center}}}}$ and ${\vec{o}}^{\,{{\rm{surround}}}}$ correspond to the experimenter-controlled velocities on the screen (ignoring observation noise), and that the perceptual reports exactly reflect the perceived variable ${\vec{v}}_{\,{{\rm{percept}}}}^{\,{{\rm{center}}}}$ (ignoring motor noise), one could estimate a per-trial modulation index to obtain a distribution over *w*_MI_ for each separation of center and surround in order to estimate means and standard deviations which unfortunately would be biased. In order to obtain the unbiased estimates reported in Fig. 3H and I, we, therefore, modeled observation noise, motor noise, and motor bias explicitly, which allowed us to infer the distribution over *w*_MI_ from the distribution over perceptual reports (see Supplementary Methods A for details). The confidence intervals around the mean and standard deviations of *w*_MI_ were estimated through bootstrapping with *p*-values being reported conservatively by counting bootstrapped samples on the side of the relevant boundary (*t*) and reporting $\frac{t+1}{N+2}$ where N is the number of bootstrap samples (5000 in our case). This conservative report is the MAP value estimated under a uniform prior, allowing us to account for the finite samples used in bootstrapping.

### Causal inference model for hierarchical motion perception

#### Generative model in a scene with two moving elements

The observed retinal velocities, ${\vec{o}}^{\,{{\rm{center}}}}$ and ${\vec{o}}^{\,{{\rm{surround}}}}$, were modeled as the true retinal velocities, ${\vec{v}}^{\,{{\rm{center}}}}$ and ${\vec{v}}^{\,{{\rm{surround}}}}$ corrupted by additive Gaussian noise with variance ${\sigma }_{\,\,{{\rm{center}}}}^{2}$ and ${\sigma }_{\,\,{{\rm{surround}}}}^{2}$, respectively (${\mathbb{I}}$ refers to a 2 × 2 identity matrix) :

2 $${\vec{o}}^{\,{{\rm{center}}}} \sim {{\mathcal{N}}}({\vec{v}}^{\,{{\rm{center}}}},{\sigma }_{{{\rm{center}}}}^{2}\,{\mathbb{I}}) \! \quad \,{\mbox{and}}\,\quad \! {\vec{o}}^{\,{{\rm{surround}}}} \sim {{\mathcal{N}}}({\vec{v}}^{\,{{\rm{surround}}}},{\sigma }_{{{\rm{surround}}}}^{2}\,{\mathbb{I}}).$$

The velocities were parameterized as two-dimensional vectors reflecting the x and y components. In order to model the inference over the different grouping trees (here, determining whether center and surround are part of the same moving group), we follow^26^ in introducing a binary (logical) variable *S *^center,surround^ ∈ {0, 1} (corresponding to the left and right side of Fig. 2B and Supplementary Fig. S2C). This allows us to write the conditional probabilities compactly as:

3 $${\vec{v}}^{\,{{\rm{center}}}} \sim {{\mathcal{N}}}({S}^{\,{{\rm{center,surround}}}}{\vec{v}}^{\,{{\rm{group}}}}+{\vec{v}}_{\,{{\rm{relative}}}}^{\,{{\rm{center}}}}+(1-{S}^{\,{{\rm{center,surround}}}}){\vec{v}}^{\,{{\rm{world}}}},{\sigma }_{\Delta }^{2}\,{\mathbb{I}})\quad \,{\mbox{and}}\,$$

4 $${\vec{v}}^{\,{{\rm{surround}}}} \sim {{\mathcal{N}}}({S}^{\,{{\rm{center,surround}}}}{\vec{v}}^{\,{{\rm{group}}}}+{\vec{v}}_{\,{{\rm{relative}}}}^{\,{{\rm{surround}}}}+(1-{S}^{\,{{\rm{center,surround}}}}){\vec{v}}^{\,{{\rm{world}}}},{\sigma }_{\Delta }^{2}\,{\mathbb{I}}).$$

where ${\sigma }_{\Delta }^{2}$ models the uncertainty in the velocity composition, e.g., due to computational noise. The prior over *S *^center,surround^ is given by *β *^center,surround^, which represents the prior probability that center and surround belong to a common structure (based on prior experience or other non-motion cues). In a scene with only two moving elements, the group velocity is inferred in the stationary world reference frame with ${\vec{v}}^{\,{{\rm{world}}}}=0$ (Fig. 1G):

5 $${\vec{v}}^{\,{{\rm{group}}}} \sim {{\mathcal{N}}}({\vec{v}}_{\,{{\rm{relative}}}}^{\,{{\rm{group}}}}+{\vec{v}}^{\,{{\rm{world}}}},{\sigma }_{\Delta }^{2}\,{\mathbb{I}}).$$

The prior over each of the relative velocities is a mixture prior of a delta function at zero and a normal distribution centered at zero

6 $${\vec{v}}_{\,{{\rm{relative}}}}^{\,{{\rm{center}}}/{{\rm{surround}}}/{{\rm{group}}}} \sim \alpha \,\delta (0)+(1-\alpha ){{\mathcal{N}}}(0,{\sigma }_{{{\rm{prior}}}}^{2}\,{\mathbb{I}})$$

where *α* represents the expectation that the (relative) motion is exactly zero (Fig. 1E, F).

#### Mapping inferred latent variables to percepts

Our model predicts that if the center is inferred to be part of the same group as the surround, then the perceived center motion corresponds to the center’s relative velocity if it is inferred to be nonzero, and to the group velocity if the center’s relative velocity is inferred to be zero. If the center is not inferred to be part of the same group as the surround, then its motion relative to the stationary world is perceived. By defining *C *^(⋅⋅⋅)^ ∈ {0, 1} to denote whether the velocity ${\vec{v}}_{\,{{\rm{relative}}}}^{\,(\cdot \cdot \cdot )}$ is zero (*C *^(⋅⋅⋅) ^= 0) or not (*C *^(⋅⋅⋅) ^= 1), we can compactly write the percept as:

7 $${\vec{v}}_{\,{{\rm{percept}}}}^{{{\rm{center}}}}={C}^{\,{{\rm{center}}}}{\vec{v}}_{\,{{\rm{relative}}}}^{\,{{\rm{center}}}}+(1-{C}^{\,{{\rm{center}}}}){S}^{\,{{\rm{center,surround}}}}{C}^{\,{{\rm{group}}}}{\vec{v}}_{\,{{\rm{relative}}}}^{\,{{\rm{group}}}}.$$

Therefore, the distribution over the observer’s percept is a mixture distribution with the mixture weights corresponding to the posterior probability of the different causal structures (characterized by possible *S*) and the different nested structures within each causal structure (characterized by possible *C*):

8 $$p({\vec{v}}_{\,{{\rm{percept}}}}^{\,{{\rm{center}}}}| \underline{\vec{o}}) 	={\sum}_{{c}_{1}=\{0,1\}}{\sum}_{{c}_{2}=\{0,1\}}{\sum}_{{c}_{3}=\{0,1\}}{\sum}_{{c}_{4}=\{0,1\}}\\ 	 p({C}^{\,{{\rm{center}}}}={c}_{1},{C}^{\,{{\rm{surround}}}}={c}_{2},{C}^{\,{{\rm{group}}}}={c}_{3},{S}^{\,{{\rm{center,surround}}}}={c}_{4}| \underline{\vec{o}})\\ 	 p({\vec{v}}_{\,{{\rm{percept}}}}^{\,{{\rm{center}}}}| \underline{\vec{o}},{C}^{\,{{\rm{center}}}}={c}_{1},{C}^{\,{{\rm{surround}}}}={c}_{2},{C}^{\,{{\rm{group}}}}={c}_{3},{S}^{\,{{\rm{center}}},{{\rm{surround}}}}={c}_{4}).$$

where for compactness we define $\underline{\vec{o}}\equiv ({\vec{o}}^{\,{{\rm{center}}}},{\vec{o}}^{\,{{\rm{surround}}}})$. For any number of moving elements, this posterior has the general form:

9 $$p({\vec{v}}_{\,{{\rm{percept}}}}^{\,{{\rm{center}}}}| \underline{\vec{o}})={\sum }_{i}^{N}{w}_{i}({\underline{\vec{o}}}_{j}){{\mathcal{N}}}({\vec{v}}_{\,{{\rm{percept}}}}^{\,{{\rm{center}}}};{\vec{\mu }}_{i}({\underline{\vec{o}}}_{j}),{\sigma }_{i}^{2}\,{\mathbb{I}})$$

where *w*_*i*_, *μ*_*i*_, and ${\sigma }_{i}^{2}$ correspond to the probability, mean, and variance associated with each of the *N* structures, respectively. For *n* moving elements, the total number of structures is bounded by $N=T(n)\,{2}^{{C}^{\max }(n)}$ wherein *T*(*n*) is the number of possible grouping trees, and ${C}^{\max }(n)=\frac{n(n+1)}{2}$ is the maximum number of nodes across all grouping trees. While this value of *N* overestimates the number of structures, the posterior is unchanged from considering the exact number of structures (see Supplementary Methods B for details). The expressions for the terms in Eq. (9) are derived in Supplementary Methods C.

The distribution over perceived velocity can be mapped onto the distribution over the perceived center direction, ${\theta }_{\,{{\rm{percept}}}}^{\,{{\rm{center}}}}$, by mapping each velocity to its corresponding direction:

10 $$p({\theta }_{\,{{\rm{percept}}}}^{\,{{\rm{center}}}}| \underline{\vec{o}})={\int}_{{\vec{v}}_{\,{{\rm{percept}}}}^{\,{{\rm{center}}}}}p(\theta | {\vec{v}}_{\,{{\rm{percept}}}}^{\,{{\rm{center}}}})p({\vec{v}}_{\,{{\rm{percept}}}}^{\,{{\rm{center}}}}| \underline{\vec{o}}).$$

Inserting Eq. (9) into (10), we can approximate the integral by an analytic form (by approximating the distribution over directions implied by a Gaussian distribution over velocities by a von Mises distribution) to get:

11 $$p({\theta }_{\,{{\rm{percept}}}}^{\,{{\rm{center}}}}| {\underline{\vec{o}}}_{j})={\sum }_{i=1}^{N}{w}_{i}(\underline{\vec{o}}){{{\mathcal{N}}}}_{{{\rm{circular}}}}\{\theta ;\hat{{\theta }_{i}}(\underline{\vec{o}}),{\kappa }_{i}\}.$$

${{{\mathcal{N}}}}_{{{\rm{circular}}}}$ represents the von Mises distribution pdf with mean parameter $\hat{{\theta }_{i}}(\underline{\vec{o}})=\arctan ({\vec{\mu }}_{i,y},{\vec{\mu }}_{i,x})$ and concentration parameter ${\kappa }_{i}={T}_{2}({\sigma }_{i}^{2}/| | {\vec{\mu }}_{i}| {| }_{2}^{2})$. This approximation was obtained by numerically simulating two-dimensional velocities under Gaussian noise and approximating the corresponding distribution over directions using a von Mises distribution. We allow for lapses in responses by adding another component to the distribution over the center direction that is a von Mises distribution characterized by *θ*_lapse_ and *κ*_lapse_.

12 $$p({\theta }_{\,{{\rm{percept}}}}^{\,{{\rm{center}}}}| \underline{\vec{o}})=\lambda {{{\mathcal{N}}}}_{{{\rm{circular}}}}(\theta ;{\theta }_{{{\rm{lapse}}}},{\kappa }_{{{\rm{lapse}}}})+(1-\lambda ){\sum }_{i=1}^{N}{w}_{i}(\underline{\vec{o}}){{{\mathcal{N}}}}_{{{\rm{circular}}}}\{\theta ;\hat{{\theta }_{i}}(\underline{\vec{o}}),{\kappa }_{i}\}.$$

which can be simplified as

13 $$p({\theta }_{\,{{\rm{percept}}}}^{\,{{\rm{center}}}}| \underline{\vec{o}})={\sum }_{i=0}^{N}w^{\prime} _{i}(\underline{\vec{o}}){{{\mathcal{N}}}}_{{{\rm{circular}}}}\left\{\theta ;\theta ^{\prime} _{i}(\underline{\vec{o}}),\kappa ^{\prime} _{i}\right\}$$

in which $w^{\prime} _{i}(\underline{\vec{o}})=(1-\lambda ){w}_{i}(\underline{\vec{o}})$, $\theta ^{\prime} _{i}=\hat{\theta}_{i}(\underline{\vec{o}})$, $\kappa ^{\prime} _{i}={\kappa }_{i}$ for *i* > 0, and $w^{\prime} _{0}=\lambda$, $\theta ^{\prime} _{0}={\theta }_{{{\rm{lapse}}}}$, $\kappa ^{\prime} _{0}={\kappa }_{{{\rm{lapse}}}}$.

#### Perceptual estimation

We consider the following four, previously proposed^26,38^, ways in which the brain may convert the posterior $p({\theta }_{\,{{\rm{percept}}}}^{\,{{\rm{center}}}}| \underline{\vec{o}})$ into a perceptual point estimate, ${\theta }_{\ {{\rm{estimate}}}}^{\ {{\rm{center}}}}({\vec{o}})$:

##### Model averaging

${\theta }_{\,{{\rm{estimate}}}}^{\,{{\rm{center}}}}$ is the mean of the joint posterior over all structures: ${\theta }_{\ {{\rm{estimate}}}}^{\ {{\rm{center}}}}({\vec{o}})={\sum }_{i=0}^{N}w^{\prime} _{i}(\underline{\vec{o}})\theta ^{\prime} _{i}(\underline{\vec{o}})$.

##### Model selection

${\theta }_{\,{{\rm{estimate}}}}^{\,{{\rm{center}}}}$ is the posterior mean over direction for the most likely structure: ${\theta }_{\ {{\rm{estimate}}}}^{\ {{\rm{center}}}}({\vec{o}})=\theta ^{\prime} _{{i}^{*}}(\underline{\vec{o}})$ where ${i}^{*}=\arg \max w^{\prime} _{i}(\underline{\vec{o}})$.

##### Structure sampling

${\theta }_{\,{{\rm{estimate}}}}^{\,{{\rm{center}}}}$ is the posterior mean over direction for a single structure sampled from the posterior over structures: ${\theta }_{\ {{\rm{estimate}}}}^{\ {{\rm{center}}}}({\vec{o}})=\theta ^{\prime} _{i}(\underline{\vec{o}})$ where probability of $\theta ^{\prime} _{i}(\underline{\vec{o}})\propto w^{\prime} _{i}(\underline{\vec{o}})$.

##### Posterior Sampling

${\theta }_{\,{{\rm{estimate}}}}^{\,{{\rm{center}}}}$ is a direction sampled from the joint posterior over all structures and directions: ${\theta }_{\ {{\rm{estimate}}}}^{\ {{\rm{center}}}}({\vec{o}}) \sim p({\theta }_{{{\rm{percept}}}}^{{{\rm{center}}}}| \underline{\vec{o}})$.

#### Predicted distribution over observer responses

The distribution over observer reports, *R*, for a set of experimenter-defined directions $\underline{\vec{\nu }}\equiv ({\vec{\nu }}_{\,{{\rm{retina}}}}^{\,{{\rm{center}}}},{\vec{\nu }}_{\,{{\rm{retina}}}}^{\,{{\rm{surround}}}})$ can be obtained by marginalizing out all possible sensory observations^26,59^ :

14 $$p(R| \underline{\vec{\nu }})={\int}_{\underline{\vec{o}}}p(R| \underline{\vec{o}})p(\underline{\vec{o}}| \underline{\vec{\nu }}).$$

We model the distribution over observer reports, *R*, as a von Mises distribution centered on ${\theta }_{\ {{\rm{estimate}}}}^{\ {{\rm{center}}}}({\vec{o}})$ allowing for a reporting bias *b* and a motor noise *κ*_*m*_:

15 $$p(R| \underline{\nu })={\int}_{\underline{\vec{o}}}{{{\mathcal{N}}}}_{{{\rm{circular}}}}(R;{\theta }_{\ {{\rm{estimate}}}}^{\ {{\rm{center}}}}({\vec{o}})+b,{\kappa }_{m})p(\underline{\vec{o}}| \underline{\vec{\nu }}).$$

Since this integral is intractable to evaluate analytically, we approximate this using Gaussian quadratures evaluated at points $\vec{{o}_{j}}$ with weights ${w}_{j}^{{{\rm{quad}}}}$:

16 $$p(R| \underline{\nu })=\mathop{\sum}_{j}{w}_{j}^{{{\rm{quad}}}}{{{\mathcal{N}}}}_{{{\rm{circular}}}}(R;{\theta }_{\ {{\rm{estimate}}}}^{\ {{\rm{center}}}}({\vec{o_j}})+b,{\kappa }_{m}).$$

The distribution over observer responses for different perceptual estimates described in the previous section are given in Supplementary Methods D.

#### Model fitting details

We obtained the maximum a posteriori (MAP; for initializing the sampler) and maximum likelihood estimate (MLE; to compute AIC) for the model parameters under weakly informative priors (details in Supplementary Tables S1, S2) using a quasi-newton Broyden-Fletcher-Goldfarb-Shanno (BFGS) unconstrained optimization procedure (fminunc in MATLAB). We obtained full posteriors over all model parameters (Supplementary Fig. S5) using generalized elliptical slice sampling^60^ which allowed us to get uncertainty estimates for all parameter estimates. We used 144 chains with 25000 samples per chain to estimate the posterior distribution over the parameters (average $\hat{R}\le 1.1$). 5000 samples were discarded from each chain as burnin and each chain was thinned by choosing every tenth sample to reduce autocorrelation between samples. The resultant 288000 samples (2000 samples in 144 chains) were used in making the posterior dependent plots (Figs. 4A, 5E–L and Supplementary Figs. S5–S7). The effective number of posterior samples^61^ for observers 1–5 were 4476, 7241, 3531, 2273, 2611 respectively (mean 4026), each greater than the recommended effective sample size of 2109 to construct a 95% credible intervals^61^.

To evaluate the absolute goodness of fit of the model, for each combination of center and surround velocities, we compared the empirical CDF from the reported directions with the corresponding model prediction. We quantified the overall match as variance explained across all conditions.

### Reporting summary

Further information on research design is available in the Nature Portfolio Reporting Summary linked to this article.

## Supplementary information

Supplementary Information

Description of Additional Supplementary Files

Supplementary Movie 1

Supplementary Movie 2

Supplementary Movie 3

Supplementary Movie 4

Supplementary Movie 5

Supplementary Movie 6

Supplementary Movie 7

Supplementary Movie 8

Supplementary Movie 9

Reporting Summary

Transparent Peer Review file

## Data Availability

Data is available at 10.17605/osf.io/f9dsg. Raw data is provided along with scripts for extracting the processed data.

## Code availability

Code is available at 10.17605/osf.io/f9dsg. Code contains both experiment code that generates the stimuli and the model code used in the analyses.
