# Supplementary material for: Hierarchical motion perception as causal inference
Source: Nat Commun. 2025 Apr 24;16:3868. doi: 10.1038/s41467-025-58797-0 (PMC12022028; doi:10.1038/s41467-025-58797-0)
Supplement: Supplementary file 2 — Description of Additional Supplementary Files [file 41467_2025_58797_MOESM2_ESM.pdf]

Supplementary Dataset

Supplementary Video 1

Johansson Illusion illustration. In the Johansson Illusion[4], two flanker dots (red) move back and forth horizontally and a center dot (green) moves diagonally. When fixating on the cross, our percept of the center dot velocity is biased to move orthogonal to the flanker dots (up and down) instead of diagonally which is the percept if the flanker dots become stationary (second half of the video).

Supplementary Video 2

Experiment 1: Stationary surround condition. Participants fixate on the center white dot are report their percept of the green dots during the third phase of the movement (indicated by fixation dot turning green). When the surround is stationary, our model predicts the percept of the green dots to be centered on the veridical velocity

Supplementary Video 3

Experiment 1: Center and Surround move in different directions. Stimuli details described in Supplementary Video 2 caption. When the center moves differently from the surround, our model predicts the percept of the green dots to be biased towards the relative velocity to the surround.

Supplementary Video 4

Experiment 1: Center and Surround move in the same direction. Stimuli details described in Supplementary Video 2 caption. When the center moves in the same direction as the surround, our model predicts the percept of the green dots to be based on a cue-combination of center and surround motion.

Supplementary Video 5

Experiment 2: Inner and Outer rings move randomly (zero motion coherence). Participants fixate on the center white dot are report their percept of the green dots. When inner and outer rings move randomly, our model predicts the percept to be centered on the veridical direction.

Supplementary Video 6

Experiment 2: Outer ring moves randomly (zero motion coherence). Stimuli details described in Supplementary Video 5 caption. When the outer ring moves randomly and inner ring moves clockwise to the center, our model predicts percepts biased towards $90°$

Supplementary Video 7

Experiment 2: Inner ring moves randomly (zero motion coherence). Stimuli details described in Supplementary Video 5 caption. When the inner ring moves randomly and outer ring moves counter-clockwise to the center, our model predicts percepts biased towards $-90°$

Supplementary Video 8

Experiment 2: All rings move coherently, center moves in a different direction than inner ring. Stimuli details described in Supplementary Video 5 caption. When the center moves in a different direction than the inner ring while the outer ring moves coherently counter-clockwise to the center, our model predicts the center is perceived in the inner rings' reference frame, resulting in a bias towards $90°$

Supplementary Video 9

Experiment 2: All rings move coherently, center moves in the same direction as the inner ring. Stimuli details described in Supplementary Video 5 caption. When the center moves in the same direction as the inner ring while the outer ring moves coherently counter-clockwise to the center, our model predicts that the center & inner ring are perceived as moving together as one group, in the reference frame of the outer ring. This predicts a perceptual bias towards $-90°$
