# Supplementary material for: Hierarchical motion perception as causal inference
Source: Nat Commun. 2025 Apr 24;16:3868. doi: 10.1038/s41467-025-58797-0 (PMC12022028; doi:10.1038/s41467-025-58797-0)
Supplement: Supplementary file 12 — Reporting Summary [file 41467_2025_58797_MOESM12_ESM.pdf]

nature portfolio

Corresponding author(s): Sabyasachi Shivkumar, Ralf Haefner

Last updated by author(s): Nov 21, 2023

Reporting Summary

Nature Portfolio wishes to improve the reproducibility of the work that we publish. This form provides structure for consistency and transparency in reporting. For further information on Nature Portfolio policies, see our Editorial Policies and the Editorial Policy Checklist.

Statistics

For all statistical analyses, confirm that the following items are present in the figure legend, table legend, main text, or Methods section.

n/a	Confirmed	
☐	☒	The exact sample size (*n*) for each experimental group/condition, given as a discrete number and unit of measurement
☒	☐	A statement on whether measurements were taken from distinct samples or whether the same sample was measured repeatedly
☐	☒	The statistical test(s) used AND whether they are one- or two-sided<br>*Only common tests should be described solely by name; describe more complex techniques in the Methods section.*
☒	☐	A description of all covariates tested
☒	☐	A description of any assumptions or corrections, such as tests of normality and adjustment for multiple comparisons
☐	☒	A full description of the statistical parameters including central tendency (e.g. means) or other basic estimates (e.g. regression coefficient) AND variation (e.g. standard deviation) or associated estimates of uncertainty (e.g. confidence intervals)
☐	☒	For null hypothesis testing, the test statistic (e.g. *F*, *t*, *r*) with confidence intervals, effect sizes, degrees of freedom and *P* value noted<br>*Give P values as exact values whenever suitable.*
☐	☒	For Bayesian analysis, information on the choice of priors and Markov chain Monte Carlo settings
☒	☐	For hierarchical and complex designs, identification of the appropriate level for tests and full reporting of outcomes
☒	☐	Estimates of effect sizes (e.g. Cohen's *d*, Pearson's *r*), indicating how they were calculated

Our web collection on statistics for biologists contains articles on many of the points above.

Software and code

Policy information about availability of computer code

Data collection: Experiment code was written using Psychtoolbox Version 3 and is available at https://osf.io/f9dsg/

Data analysis: Custom code in Matlab (version 2021a) and the code is available at https://osf.io/f9dsg/ was used for data analysis

For manuscripts utilizing custom algorithms or software that are central to the research but not yet described in published literature, software must be made available to editors and reviewers. We strongly encourage code deposition in a community repository (e.g. GitHub). See the Nature Portfolio guidelines for submitting code & software for further information.

Data

Policy information about availability of data

All manuscripts must include a data availability statement. This statement should provide the following information, where applicable:

- Accession codes, unique identifiers, or web links for publicly available datasets
- A description of any restrictions on data availability
- For clinical datasets or third party data, please ensure that the statement adheres to our policy

All data is available at https://osf.io/f9dsg/

Research involving human participants, their data, or biological material

Policy information about studies with human participants or human data. See also policy information about sex, gender (identity/presentation), and sexual orientation and race, ethnicity and racism.

Reporting on sex and gender	Sex and Gender information were not collected for privacy as they were not relevant for our study and our finding are applicable across sex and gender
Reporting on race, ethnicity, or other socially relevant groupings	No categorization based on race, ethnicity or other socially relevant groupings was used in our study as they were not relevant for our studies and our findings are applicable across these categories.
Population characteristics	No specific covariate relevant population characteristic was applicable for our study
Recruitment	Participants were recruited using email advertisements sent to appropriate university mailing lists
Ethics oversight	Experiments were approved by the Office of Human Subject Protection (OHSP) at the University of Rochester (IRB number 0003909)

Note that full information on the approval of the study protocol must also be provided in the manuscript.

Field-specific reporting

Please select the one below that is the best fit for your research. If you are not sure, read the appropriate sections before making your selection.

☐ Life sciences ☒ Behavioural & social sciences ☐ Ecological, evolutionary & environmental sciences

For a reference copy of the document with all sections, see nature.com/documents/nr-reporting-summary-flat.pdf

Behavioural & social sciences study design

All studies must disclose on these points even when the disclosure is negative.

Study description	The study is a quantitative behavioral study. Participants were shown moving dot stimuli in which colored dots moved in different directions and the participants were asked to report the direction of movement of the green dots by rotating a dial
Research sample	The participants were students from University of Rochester and were chosen randomly using email advertisements to University mailing lists. Demographic information were not collected as they are not relevant for our findings.
Sampling strategy	We used a voluntary response sample as participants were recruited using email advertisements sent to appropriate University mailing lists. Since our predictions were tested on an individual participant level, we asked each participant to perform three sessions and collected responses from 5 participants in Experiment 1 and 10 (1 excluded using specified exclusion criterion) participants in Experiment 2.
Data collection	Participants performed the experiment at a psychophysics lab booth in University of Rochester where their eye movements were recorded using Eyelink eye tracker and they indicated their responses using a dial.
Timing	Data for Experiment 1 was collected between 08/03/2019 and 10/29/2019<br>Data for Experiment 2 was collected in two cohorts between: (1) 02/28/2020 and 03/14/2020 and (2) 03/20/2021 and 12/01/2021 (gap between cohorts due to shutdown in COVID19 pandemic)
Data exclusions	1/10 observers in Experiment 1 were excluded based on their large response variability (standard deviation greater than 30 deg.)
Non-participation	No participants dropped out from participation
Randomization	The participants were students from University of Rochester and were chosen randomly using email advertisements to University mailing lists.

Reporting for specific materials, systems and methods

We require information from authors about some types of materials, experimental systems and methods used in many studies. Here, indicate whether each material, system or method listed is relevant to your study. If you are not sure if a list item applies to your research, read the appropriate section before selecting a response.

Materials & experimental systems

n/a	Involved in the study
☒	☐ Antibodies
☒	☐ Eukaryotic cell lines
☒	☐ Palaeontology and archaeology
☒	☐ Animals and other organisms
☒	☐ Clinical data
☒	☐ Dual use research of concern
☒	☐ Plants

Methods

n/a	Involved in the study
☒	☐ ChIP-seq
☒	☐ Flow cytometry
☒	☐ MRI-based neuroimaging

Plants

Seed stocks

Report on the source of all seed stocks or other plant material used. If applicable, state the seed stock centre and catalogue number. If plant specimens were collected from the field, describe the collection location, date and sampling procedures.

Novel plant genotypes

Describe the methods by which all novel plant genotypes were produced. This includes those generated by transgenic approaches, gene editing, chemical/radiation-based mutagenesis and hybridization. For transgenic lines, describe the transformation method, the number of independent lines analyzed and the generation upon which experiments were performed. For gene-edited lines, describe the editor used, the endogenous sequence targeted for editing, the targeting guide RNA sequence (if applicable) and how the editor was applied.

Authentication

Describe any authentication procedures for each seed stock used or novel genotype generated. Describe any experiments used to assess the effect of a mutation and, where applicable, how potential secondary effects (e.g. second site T-DNA insertions, mosiacism, off-target gene editing) were examined.
