# Supplementary material for: Hierarchical motion perception as causal inference
Source: Nat Commun. 2025 Apr 24;16:3868. doi: 10.1038/s41467-025-58797-0 (PMC12022028; doi:10.1038/s41467-025-58797-0)
Supplement: Supplementary file 13 — Transparent Peer Review file [file 41467_2025_58797_MOESM13_ESM.pdf]

nature portfolio

Peer Review File

Hierarchical motion perception as causal inference

Corresponding Author: Dr Sabyasachi Shivkumar

This manuscript has been previously reviewed at another journal. This document only contains information relating to versions considered at Nature Communications.

This file contains all reviewer reports in order by version, followed by all author rebuttals in order by version.

Version 0:

Reviewer comments:

Reviewer #1

(Remarks to the Author)
In this paper, the authors present modeling and experimental data of human motion inference. The key idea, building on earlier proposals, is that people decomposing complex motions into hierarchies of simpler relative motions. They find quantitative support for a particular implementation of this model. In addition to a new experimental paradigm for testing model predictions, a principal theoretical innovation of the paper is the assumption (supported by experimental results) that human priors place extra probability mass on stationary reference frames.

Overall, I thought the paper was clearly written and provides a valuable contribution to the literature on human motion perception.

Major:

The treatment of previous literature could be improved, particularly with regard to past modeling.
- p. 1: Papers by Bill et al. (2020, 2022) are cited to support the claim that "quantitative empirical tests of these models are based on explicit questions like 'Do you perceive structure A or B?'". Note that the 2022 paper is purely theoretical, and the 2020 paper does not ask these explicit questions (it uses tracking and prediction tasks). It's also asserted that explicit questions about structure are prone to decision biases, but this is not explained; Noel et al. (2022) is cited, but it's not clear how their data pose a fundamental problem for the intepretation of explicit measures in the tasks studied (for example) by Gershman et al. (2016) and Yang et al. (2021). In any case, I agree that we should use both explicit and implicit measures. I just feel that this kind of vague/inaccurate criticism of past literature is unhelpful. My last small gripe with this sentence is that it makes it seem like the only quantitative tests of computational Gestalt models have been in the domain of motion perception. This neglects the extensive quantitative work by people like Jacob Feldman, Manish Singh, and others who have studied Gestalt phenomena in other domains (e.g., contour and shape perception, figure-ground segregation, etc.).
- p. 13: The authors seem to be arguing that motion structure and velocity were not jointly inferred in previous work. This is incorrect. Both Gershman et al. (2016) and Bill et al. (2022) do this (I'm not sure what "long time scales" means in this paragraph).
- Generally speaking, I felt that the authors have not really acknowledged the fact that some of their theoretical machinery (e.g., a Gaussian process defined over a motion tree) has been proposed before. This prevents them from clarifying what is really novel about their contribution.

Descriptive and inferential statistics for the experimental results are lacking. It wasn't clear whether, in Experiment 1, the inferred motion direction for small center directions really differed getween stationary and moving surrounds. This requires a more rigorous statistical analysis. The authors should also be explicit about what statistical tests they are performing (e.g., last paragraph on p. 6).

One general point about both experiments: I'd like to see more direct comparison between models and data, side-by-side (or superimposed).

Minor:

p. 1: The first paragraph refers to the Johansson illusion, but not all readers may be familiar with this. I think a visual aid would be helpful.

p. 2: "approximately rigid objects who in turn" -> "approximately rigid objects that in turn"

Fig 2 caption: "Johansson (1950)" -> "(Johansson, 1950)"

Fig 4 caption: "is show" -> "is shown"

Fig 4B: why is AIC referred to as a "relative log-likelihood"? It includes a complexity penalty, so it can't straightforwardly be interpreted in this way.

Fig 5 caption: "as no influence" -> "has no influence"

p. 12: "patchmoving" -> "patch moving"

Make sure to state what the error bars show in the figure captions.

It was unclear to me how Eq. 11 was derived from Eqs. 9 and 10. Where did the circular normal come from?

Reviewer #2

(Remarks to the Author)
This article is a careful look at causal inference wherein both object identities (integration and segmentation) and their state of motion are inferred based on apparent motion of groups of points.

The joint inference framework explains many motion perception biases due to the different integration and segmentation of the target and reference frame wrt which the motion is perceived. Several elements of the model are investigated in two experiments; the low velocity prior and the hierarchical nature of the inference, and the form of approximate inference.

This is an extremely thorough, innovative an insightful paper that meets the highest standards of rigor. I'm not really sure how it could be made even better, but I do have a have very minor suggestions:

Even though the stimuli motion are described in the methods, I'm wondering if it couldn't be possible to illustrate the moving experimental stimuli in one of the early diagram to show the range of motion; for example, start, midpoint and final (one period of oscillation as the case may be) or a blurred stimuli over the whole experiment (long exposure).

For the results presented in Fig 5, it is much less intuitively and visually clear what the model predicts. I understand from the text that one expects the reference frame to shift between the inner circle and the outer circle when the difference in velocity between the innermost group varies. However, I'm more confused about the partial effect -- why the experimental results visually seem to be biased toward (-30, 30) and not (-90, 90) as described in the text. I'm not sure why this biasing appears weaker than in Fig 3 overall (or is it?). Also the (different?) subject colors is not indicated on Fig. 5. Overall, I feel that Fig 5B would be better served by separating and expanding the model prediction element into another panel.
Typos:
abstract: double closing quotation mark.
l. 239 patchmoving -> patch moving
Fig 4 caption: ... is show -> ..is shown

Reviewer #3

(Remarks to the Author)
In this manuscript, the authors extend the Bayesian Causal Inference framework (earlier proposed in the field of multisensory integration), and adapt it to the specific case of visual motion perception in the context of multiple motion cues and a hierarchical structure. This is an original and well-grounded idea which provides a valuable contribution to a field of research (the one on motion integration vs segmentation and in general the one on complex visual motion) which has strongly guided our understanding of fundamental brain computations, in the past, but which has recently received less attention, unfortunately. Importantly, this work pushes forward the idea that causal inference is a very general computation, and it supports this claim with sound experimental evidence and detailed model comparison. The manuscript is overall clear and well written, although it is very dense in terms of complex information and I think that some points deserve an effort of clarification, especially regarding some figures, the rationale of some choices, and the discussion.
I list these attention-deserving points below.

Rationale and details of the motion perception tasks:
- Dot groups are segmented by color, a possible biasing factor for causal inference? in a study investigating the deep nature of structured motion perception and the of segmentation/integration processes it was a bit disappointing to see model predictions tested using stimuli where color provides a potentially strong cue about the belongingness of dots to motion structures. The authors could have designed a different task where the structures' segregation is only based on motion cues… Yet, given the results one can conclude a posteriori that the bias (presumably in favor of a stronger segregation of

different color-based groups) was not dramatic. The authors might want to comment about this point
- Moving stimuli are presented at 5 degrees eccentricity: the authors should explain the reason of this choice. Would the inferential computations work differently in the central visual field? Are there specific hypotheses about this?
Also, the dots motion speed is unusually small (1 /s): is there a specific reason? Finally, the rationale of the initial “back and forth” motion of the stimuli in experiment 1 is not clear to me.
- As a matter of fact, the proposed theory is tested on motion direction rather than speed estimation: this is not a problem as the results are very meaningful for the general framework anyway, but this specificity should be acknowledged, because some differences exist between speed and direction perception (e.g. the robustness to sensory noise, and others) and could for instance affect the transition from integration to segregation in more complex displays.

Role of the zero and low-speed Prior (with respect to the previously established low-speed Prior): the results and model fitting provided in this study are very convincing and clearly support the relevance of the mass associated to purely static objects in the Prior. However, this novel and interesting (and a posteriori one could even add intuitive!) result should be further discussed, in my view. For instance Stocker and Simoncelli (2006) found that motion perception data were better explained by a slowly decreasing Prior (with heavier tales than a Gaussian): isn’t their finding contradictory with respect to the present one, where a lot of mass in zero-velocity is needed? Could the different experimental contexts (fine speed discrimination of low contrast single moving stimuli in their case; direction estimation of structured multi-dots motion here) explain why different Prior shapes could satisfactorily explain the two sets of results? Or is this after all not a relevant difference?

Causal vs hierarchical inference for motion perception: a more expanded illustration on the different predictions (with examples of specific differences, rather than only the AIC comparison of figure 4B) between Bill et al. model vs the present one in the context of motion integration/segmentation would further strengthen the paper.

Figure 4.C-F : the illustration of the different structures is confusing, in particular I find that there are too many arrows and the role of each graphic element in the figures is not explained either in the caption or in the main text and it is actually not intuitive at all. I recommend simplifying the graphics, or, alternatively, to explain the match between the graphic plots and the theoretical structures more in detail in the text (but I would favor the first option, to simplify).

Discussion, Lines 303-308: the reasoning in this paragraph is not clear and possibly misleading: the cited study by Chopin and colleagues focuses on binocular rivalry with perceptually biased stimuli and it seems somewhat risky to draw a parallel between the fusion/rivalry phenomenon there and the motion integration/segregation here. Similarly, the manipulation of the serial effects considered in the study by Cicchini and collaborators do not have a straightforward correspondence with the manipulation in this manuscript. Overall, I would say that the context of those experiments and the present one is not comparable at this stage, or not in an obvious way: in order to make the comparison more plausible one could consider different manipulations of the perceived speed of the surround dots (e.g. obtained by lowering their contrast or another extrinsic experimental variable)… Finally, I do not really see how the arguments discussed here would support two different models of inference for perception along the dorsal and ventral streams. This claim seems far-fetched, and I would urge the authors to provide in-depth and clear arguments in favor of this conclusion, or to omit it.

Minor points:
- The seminal paper by Ernst and Banks (2002) is cited sometimes in a not fully appropriate context: at line 115 for instance a reference to a more general work about the combination of independent noisy sensory cues would be more appropriate (e.g. the book “Sensory cue integration”, Oxford University Press, or even an earlier reference among the theoretical studies by Youille, or similar ones…)
- Discussion, Lines 298-302 (starting with “A corollary to this result…”): this paragraph is not clear at all to me. It is maybe only my problem, but I guess that it should be more explicit about the logical step
- I spotted few small typos here and there:
Caption of Figure 5, “h” missiong in “has”
Line 278, “our our”
Line 377, symbol of degree missing
Line 416, “s” missing
Line 461, “n” missing

Version 1:

Reviewer comments:

Reviewer #1

(Remarks to the Author)
The authors have done a good job addressing my comments. I have a few easily addressed comments about the discussion of past literature:
- "use priors that are not justified by our knowledge about the world but instead by computational tractability (Jäkel et al., 2016)". I don't see how this statement how this statement is valid. First, the model in that paper was indeed motivated by a claim that hierarchical reference frames reflect 'knowledge of the world' imputed to human observers. Second, I think the authors are referring to the fact that in the 2016 paper a Gaussian process assumption was invoked, which has convenient

analytical properties (under a superposition assumption, the latent functions can be marginalized). This assumption could be criticized (indeed the original authors criticized it themselves), but it was not made *purely* for computational convenience. This was a substantive hypothesis about how humans analyze hierarchical motion structure, and it provided a good qualitative and quantitative match to the available data. I don't think it's necessary to take vague pot-shots at past work in order to motivate the current work. [Incidentally, I think maybe the authors meant to cite Gershman et al., 2016 here?]
- Explicit questions about structure identity used in past work are criticized as being prone to decision biases. While I understand this is possible in principle, I think it's encumbent on the authors, if they really want to make this point, to show how previous results could have arisen artifactually from decision biases.
- "Other studies (Bill et al. 2020) employ a motion prediction task with reports in the retinotopic reference frame. This design avoids the problem of decision biases by inferring the structure from reports about a different question. However, while successful in identifying the structure, this design does not address how the motion is perceived given the inferred structures." I don't understand this. Isn't the motion prediction task precisely getting at how motion is perceived given the inferred structures?
- The discussion of sampling vs. MAP reports was confusing to me, because Bill et al. (2020) assumed MAP inference over structure and then sampling of perceptual reports (this might count as a 'flexible' mapping). It's important to distinguish sampling for structure inference and sampling for perceptual reports.

(Remarks on code availability)

Reviewer #2

(Remarks to the Author)
No further comments. I congratulate the authors on this impressive work.

(Remarks on code availability)

Reviewer #3

(Remarks to the Author)
The authors have very carefully and thoughtfully taken into account the reviewers' comments. The manuscript has further improved in quality in my view. Very nice work!

(Remarks on code availability)

Open Access This Peer Review File is licensed under a Creative Commons Attribution 4.0 International License, which permits use, sharing, adaptation, distribution and reproduction in any medium or format, as long as you give appropriate credit to the original author(s) and the source, provide a link to the Creative Commons license, and indicate if changes were made.
In cases where reviewers are anonymous, credit should be given to 'Anonymous Referee' and the source.

The images or other third party material in this Peer Review File are included in the article's Creative Commons license, unless indicated otherwise in a credit line to the material. If material is not included in the article's Creative Commons license and your intended use is not permitted by statutory regulation or exceeds the permitted use, you will need to obtain permission directly from the copyright holder.
To view a copy of this license, visit https://creativecommons.org/licenses/by/4.0/

We thank the reviewers for their insightful comments and provide a point-by-point response. We also quote the changes made in the manuscript in red.

Reviewer #1 (Remarks to the Author):

In this paper, the authors present modeling and experimental data of human motion inference. The key idea, building on earlier proposals, is that people decomposing complex motions into hierarchies of simpler relative motions. They find quantitative support for a particular implementation of this model. In addition to a new experimental paradigm for testing model predictions, a principal theoretical innovation of the paper is the assumption (supported by experimental results) that human priors place extra probability mass on stationary reference frames.

Overall, I thought the paper was clearly written and provides a valuable contribution to the literature on human motion perception.

Thank you for the positive evaluation and your helpful comments.

Major:

The treatment of previous literature could be improved, particularly with regard to past modeling.
- p. 1: Papers by Bill et al. (2020, 2022) are cited to support the claim that "quantitative empirical tests of these models are based on explicit questions like 'Do you perceive structure A or B?'". Note that the 2022 paper is purely theoretical, and the 2020 paper does not ask these explicit questions (it uses tracking and prediction tasks).

It's also asserted that explicit questions about structure are prone to decision biases, but this is not explained; Noel et al. (2022) is cited, but it's not clear how their data pose a fundamental problem for the intepretation of explicit measures in the tasks studied (for example) by Gershman et al. (2016) and Yang et al. (2021). In any case, I agree that we should use both explicit and implicit measures. I just feel that this kind of vague/inaccurate criticism of past literature is unhelpful.

Thank you for pointing out these inaccuracies, and for encouraging us to unpack some of our criticism. We have now corrected, and made more precise, the context for each of these citations in the Introduction and Discussion:

In the introduction, we have expanded the details of how each line of works builds on top of another while softening the criticisms. We have also clarified the shortcomings of asking for an explicit report.

Changed lines 21-32

Yet, existing Bayesian accounts of motion perception are either formulated in a purely retinotopic reference frame (Weiss et al. 2002; Stocker & Simoncelli, 2006) or use priors that are not justified by our knowledge about the world but instead by computational tractability (Jäkel et al., 2016). Furthermore, quantitative empirical tests of some models in the motion

domain are based on explicit questions about structure identity like ``Do you perceive structure A or B?'' (Gershman et al. 2016; Yang et al. 2021) which are known to be susceptible to decision biases which need to be estimated separately (Noel et al. 2022). These biases could arise for example from categories presented according to a different distribution than observers' beliefs. Other studies (Bill et al. 2020) employ a motion prediction task with reports in the retinotopic reference frame. This design avoids the problem of decision biases by inferring the structure from reports about a different question. However, while successful in identifying the structure, this design does not address how the motion is perceived given the inferred structures.

In the discussion, we have further compared similarities of previous work to our work before pointing out differences.

Changed lines 332-343
Our work was strongly inspired by Gershman et al. (2016) who embedded the vector decomposition idea of Johansson (1950) in a Bayesian inference framework and extended it both hierarchically and spatially.
We built upon their model in three ways: first, we changed the structure of the model so that inference only required local computations. Second, this allowed us to define prior distributions over the relative velocities, and to use known physics to inform them by including a delta-component at zero. This prior over velocities also implied a prior over causal structures that differed from: (a) the Chinese Restaurant Process (CRP) prior assumed by Gershman et al. (2016) that placed elements in a group in proportion to its size, and (b) from related works (Bill et al., 2020, 2022) that used a Jeffrey's prior over motion strength to penalize the levels of hierarchy (Bill et al., 2022, 2020). Finally, we allowed for a flexible mapping from posteriors over structures and velocities to perceptual reports. Our data strongly constrained this mapping to correspond to sampling, different from the MAP inference over structures assumed by related prior works (Gershman et al., 2016; Bill et al., 2020; Yang et al., 2021; Bill et al., 2022).

Hopefully, these additions do a better job of placing our work in the context of earlier work.

My last small gripe with this sentence is that it makes it seem like the only quantitative tests of computational Gestalt models have been in the domain of motion perception. This neglects the extensive quantitative work by people like Jacob Feldman, Manish Singh, and others who have studied Gestalt phenomena in other domains (e.g., contour and shape perception, figure-ground segregation, etc.).

Thank you for these suggestions: we already cited Feldman's work but have now made it clear that our statement about quantitative tests only applies to motion models. We would be happy to cite any relevant papers by Singh but we could only find relevant VSS abstracts.

Changed lines 15-18
Previous work has made some progress in mathematically formalizing this elusive concept of a Gestalt: first in information-theoretic terms (Pomerantz and Kubovy, 1986; Restle, 1979), and more recently in closely-related Bayesian terms (Froyen et al., 2015; Feldman, 2009; J¨akel et al., 2016).

- p. 13: The authors seem to be arguing that motion structure and velocity were not jointly inferred in previous work. This is incorrect. Both Gershman et al. (2016) and Bill et al. (2022) do this (I'm not sure what "long time scales" means in this paragraph).

We apologize for the confusing formulation which we have now made more precise by stating that both Gershman et al. (2016) and Bill et al. (2022) assumed MAP inference over structures and velocity averaging within the most likely structure.
We double-checked with the authors of Bill et al. (2022) that "v_perceived averaged over the last 10s of each trial" did not imply model averaging, but only averaging within one structure. Their lambda-parameter representing the structure was inferred via EM to converge to the MAP, and only represented different structures on a time scale longer than 10s, with switches only due to observation noise (not sampling from the posterior) and the averaging only served to reduce noise

Changed lines 341-343
Our data strongly constrained this mapping to correspond to sampling, different from the MAP inference over structures assumed by related prior works (Gershman et al., 2016; Bill et al., 2020; Yang et al., 2021; Bill et al., 2022)

.

- Generally speaking, I felt that the authors have not really acknowledged the fact that some of their theoretical machinery (e.g., a Gaussian process defined over a motion tree) has been proposed before. This prevents them from clarifying what is really novel about their contribution.

Thank you for this comment: this is definitely not the impression that we wanted to create. We have now added an entirely new paragraph (3rd paragraph of Discussion) dedicated to describing in detail how our model builds on previous work and how it deviates from it, and goes beyond it. We copy it below:

Changed lines 332-348
Our work was strongly inspired by Gershman et al. (2016) who embedded the vector decomposition idea of Johansson (1950) in a Bayesian inference framework and extended it both hierarchically and spatially.
We built upon their model in three ways: first, we changed the structure of the model so that inference only required local computations. Second, this allowed us to define prior distributions over the relative velocities, and to use known physics to inform them by including a delta-component at zero. This prior over velocities also implied a prior over causal structures that differed from: (a) the Chinese Restaurant Process (CRP) prior assumed by Gershman et al. (2016) that placed elements in a group in proportion to its size, and (b) from related works (Bill et al., 2020, 2022) that used a Jeffrey's prior over motion strength to penalize the levels of hierarchy (Bill et al., 2022, 2020). Finally, we allowed for a flexible mapping from posteriors over structures and velocities to perceptual reports. Our data strongly constrained this mapping to correspond to sampling, different from the MAP

inference over structures assumed by related prior works (Gershman et al., 2016; Bill et al., 2020; Yang et al., 2021; Bill et al., 2022).

Descriptive and inferential statistics for the experimental results are lacking. It wasn't clear whether, in Experiment 1, the inferred motion direction for small center directions really differed between stationary and moving surrounds. This requires a more rigorous statistical analysis. The authors should also be explicit about what statistical tests they are performing (e.g., last paragraph on p. 6).

We apologize for not placing the statistics right after our integration claim. We have now weakened the initial statement based on visual inspection to "appear biased" and have moved the description of the quantification and statistical tests we perform to the next paragraph. There we provide p-values for integration for both the group ($p<0.001$) and the individual observers ($p<0.05$ for 4 out of 5 observers).

Changed lines 178-185

The mean modulation index, averaged across observers (Figure 3H), is negative for a center direction of 2.5◦, indicating integration ($p < 0.001$ for the group, $p < 0.05$ individually for 4 out of 5 observers based on 5000 bootstrapped samples). For larger separations (greater than 10◦), the average and individual mean modulation indices are positive indicating segmentation for larger separations ($p < 0.001$, based on 5000 bootstrapped samples). For larger separations). The standard deviation of modulation index, averaged across observers (Figure 3F) is largest for intermediate separations ($p < 0.01$, based on 5000 bootstrapped samples). For larger separations for the difference in the standard deviations of the modulation index at 5◦ and at 2.5◦ and 45◦) indicating a higher variability due to uncertainty over causal structures, in agreement with our model predictions.

At the same time we note that looking at the raw data, the integration effect is not obviously significant. This is because in Experiment 1 (and all similar experiments in the literature), the effect size is very small, and competing with various noise sources, in particular motor noise. The modulation index combines data from all conditions, including stationary surround, to estimate these noise sources, and is thereby able to distinguish integration from reporting retinal motion with higher power.

We therefore followed Rutherford's dictum "If your result depends on statistics then you need a better experiment" and designed Experiment 2 partly in order to maximize the integration effect size and make it plainly visible to the naked eye. See Fig S16 for the raw data for Experiment 2. Note that errorbars represent 95% confidence intervals around the median, and none of them for the smallest two separations of 0 and 3deg overlap with zero.

One general point about both experiments: I'd like to see more direct comparison between models and data, side-by-side (or superimposed).

We already show each model fit superimposed with the data in both Fig 3 and in the supplementary information (Fig. S3). We have slightly expanded the description of the fits in Fig 3 to make it less likely to be overlooked.

(Apologies if we misunderstood the reviewer’s comment.)

Changed Figure 3 caption

(B-G) The overlaid violin plots show the model predictions (not data distributions). One model was fit jointly on all data for each observer

Minor:

p. 1: The first paragraph refers to the Johansson illusion, but not all readers may be familiar with this. I think a visual aid would be helpful.

We have now added a video in the supplementary information illustrating the Johansson illusion.

Added supplementary video

p. 2: "approximately rigid objects who in turn" -> "approximately rigid objects that in turn"

Corrected

Fig 2 caption: "Johansson (1950)" -> "(Johansson, 1950)"

Corrected

Fig 4 caption: "is show" -> "is shown"

Corrected

Fig 4B: why is AIC referred to as a "relative log-likelihood"? It includes a complexity penalty, so it can't straightforwardly be interpreted in this way.

Relative likelihood when applied to models instead of different parameter values is defined in terms of the difference in AIC. However, to avoid confusion, we have now replaced relative likelihood with ΔAIC both in Figure 4 and Supplementary Figure S7.

Changed Figure 4B and S7

Fig 5 caption: "as no influence" -> "has no influence"

Corrected

p. 12: "patchmoving" -> "patch moving"

Corrected

Make sure to state what the error bars show in the figure captions.

Corrected

It was unclear to me how Eq. 11 was derived from Eqs. 9 and 10. Where did the circular normal come from?

This approximation was obtained by numerically simulating two-dimensional velocities under Gaussian noise and approximating the corresponding distribution over directions using a von Mises distribution. We have now added this to the main text.

Changed lines 521-523
This approximation was obtained by numerically simulating two-dimensional velocities under Gaussian noise and approximating the corresponding distribution over directions using a von Mises distribution.

Reviewer #2 (Remarks to the Author):

This article is a careful look at causal inference wherein both object identities (integration and segmentation) and their state of motion are inferred based on apparent motion of groups of points.

The joint inference framework explains many motion perception biases due to the different integration and segmentation of the target and reference frame wrt which the motion is perceived. Several elements of the model are investigated in two experiments; the low velocity prior and the hierarchical nature of the inference, and the form of approximate inference.

This is an extremely thorough, innovative an insightful paper that meets the highest standards of rigor. I'm not really sure how it could be made even better, but I do have a have very minor suggestions:

Thank you for the positive assessment!

Even though the stimuli motion are described in the methods, I'm wondering if it couldn't be possible to illustrate the moving experimental stimuli in one of the early diagram to show the range of motion; for example, start, midpoint and final (one period of oscillation as the case may be) or a blurred stimuli over the whole experiment (long exposure).

Thank you for this suggestion. We have now modified Fig. 3A to include an illustration of the time course of a trial, as well as the velocity of various dots using arrows. We have also now included videos of the stimuli in the supplementary information.

Changed Figure 3A and added supplementary video

For the results presented in Fig 5, it is much less intuitively and visually clear what the model predicts. I understand from the text that one expects the reference frame to shift between the inner circle and the outer circle when the difference in velocity between the innermost group

varies. However, I'm more confused about the partial effect -- why the experimental results visually seem to be biased toward (-30, 30) and not (-90, 90) as described in the text. I'm not sure why this biasing appears weaker than in Fig 3 overall (or is it?). Also the (different?) subject colors is not indicated on Fig. 5. Overall, I feel that Fig 5B would be better served by separating and expanding the model prediction element into another panel.

Thank you for these helpful comments. We have now simplified the figure layout to indicate that (a) -90 degrees is the relative velocity of any weighted combination of the center and inner ring to the outer ring, and (b) 90 degrees is the relative velocity of the center to the inner ring. These are designed to match the predicted percept under two boundary structures under the model, (a) the center & inner ring group is perceived in the reference frame formed by the outer ring alone, and (b) the center is perceived in the reference frame formed by the inner ring alone. The model has many other structures which predict intermediate percepts and the average response over many trials will be an average over the percepts predicted by different model structures. This explains the data whose average asymptotes around +/- 30deg. In line with this explanation, the per-trial data shown in Supplemental Figure 19 clearly shows a much wider range of per-trial perceptual reports, including the full range of -90deg to +90deg.

Ideally, we'd like to fit the full model to data from Experiment 2 as we did for Experiment 1 but unfortunately, because of the combinatorial explosion of possible structures (264 for just 3 levels, see a subset in Figures S11-S16), this is very expensive and beyond the scope of this paper. However, we can make model predictions under two simplifying assumptions:

(a) We only consider structures in which the relevant reference frame coincides with one of the moving elements. This allows us to ignore structures in which the reference frame moves at an intermediate velocity (as e.g. in Fig 4, Structure 4).
(b) We assume that the sensory uncertainty of the inner ring velocity is much lower than the uncertainty related to the center (since it is smaller than the inner ring), and the sensory uncertainty of the outer ring is much lower than the inner ring (since it is smaller than the outer ring). Under these assumptions, the group velocity can be approximated by the velocity of the most reliable element forming the group.

With these simplifying assumptions, the model contains 44 structures (as opposed to 264) which allow us to understand the model predictions qualitatively (the remaining structures only predict intermediate percepts to those predicted by the simplified model). We show these 'pure' causal structures and the corresponding predicted posterior probabilities in Figures S11-S16. We now show the predictions of the simplified model in Fig. S10 and describe the model in Supplementary section E.

Changed Figure 6B and added Supplementary Information section E and figures S10-S16

Typos:
abstract: double closing quotation mark.
l. 239 patchmoving -> patch moving
Fig 4 caption: ... is show -> ..is shown

We thank the reviewer for pointing out the typos and have now corrected them in the revised manuscript.

Reviewer #3 (Remarks to the Author):

In this manuscript, the authors extend the Bayesian Causal Inference framework (earlier proposed in the field of multisensory integration), and adapt it to the specific case of visual motion perception in the context of multiple motion cues and a hierarchical structure. This is an original and well-grounded idea which provides a valuable contribution to a field of research (the one on motion integration vs segmentation and in general the one on complex visual motion) which has strongly guided our understanding of fundamental brain computations, in the past, but which has recently received less attention, unfortunately. Importantly, this work pushes forward the idea that causal inference is a very general computation, and it supports this claim with sound experimental evidence and detailed model comparison. The manuscript is overall clear and well written, although it is very dense in terms of complex information and I think that some points deserve an effort of clarification, especially regarding some figures, the rationale of some choices, and the discussion.
I list these attention-deserving points below.

We thank the reviewer for this positive assessment, and the constructive feedback below!

Rationale and details of the motion perception tasks:
- Dot groups are segmented by color, a possible biasing factor for causal inference? in a study investigating the deep nature of structured motion perception and the of segmentation/integration processes it was a bit disappointing to see model predictions tested using stimuli where color provides a potentially strong cue about the belongingness of dots to motion structures. The authors could have designed a different task where the structures' segregation is only based on motion cues… Yet, given the results one can conclude a posteriori that the bias (presumably in favor of a stronger segregation of different color-based groups) was not dramatic. The authors might want to comment about this point

We thank the reviewer for raising this point. During piloting, we started with stimuli where center and surround had the same color. Unfortunately, observers found it very hard to distinguish center from surround, presumably due to crowding effects, making it hard for them to reliably report the center direction. We therefore used different colors to avoid noise in the data due to interference effects. Initially we were worried that using different colors would make it less likely for the observer to infer both center and surround to belong to the same causal structure, and as a result diminish the effect of the surround on perception which we wanted to study. However, that did not turn out to be the case. In future studies in would be interesting to systematically study the effect of color on grouping in motion perception: our results suggest that the effect will be minimal. We now address this points in the Discussion.

Changed lines 374-382
In our stimuli, center dots had a different color from the surround in order to minimize observer confusion about which motion direction to report. This difference in color might have increased the probability of segmenting the center from the surround (we found

anecdotal evidence during pilot sessions that separating center from surround was difficult when both were of the same color making reporting the center direction hard). Using our experimental paradigm, and model-based analysis, the effect of color, as well as the effect of other cues like spatial relationships, disparity, etc. can be quantitatively studied, and related to model parameters like the prior probability of inferred the same motion, or the same causal structure. Furthermore, since our work was entirely based on the perception of motion directions, future work should also test our model predictions for speed perception.

- Moving stimuli are presented at 5 degrees eccentricity: the authors should explain the reason of this choice. Would the inferential computations work differently in the central visual field? Are there specific hypotheses about this?

There are two reasons why we decided to place the stimuli at the periphery:

a) Presenting moving dots at fixation, makes it very difficult not to track the dots thereby changing the observed retinal motion. While this could be modeled as self motion in the model, for simplicity and control, we presented dots in the periphery and ensured fixation using an eye-tracker to prevent eye movements and tracking.
b) Performing the experiment in the periphery makes it easier for us and others to build on our results in order to study the neural basis of motion perception in e.g. area MT where neurons with peripheral receptive fields are easier to access and to study than those with foveal receptive fields.

While there are no differences between foveal and peripheral visual processing in our model (other than lower sensory uncertainty in the fovea, of course), studying the effects of tracking dots would be interesting future work. Previous studies (Freeman et al. 2010) have explored such biases due to pursuit (Fillehne illusion, in which stationary objects move in opposite direction of eye movements) as a consequence of the brain removing its eye movement component. In our model, such effects can be incorporated as self motion in order to make testable predictions for these effects and how they interact with the grouping effects in our experiment. We now clarify this in the discussion

Added lines 382-385

We also presented our stimuli in the periphery to prevent observers from tracking the dots, resulting in previously studied biases (Freeman et al. 2010), and to facilitate future neurophysiological studies in areas like MT. Future work can study the empirical influence of eye movements and incorporate them in our model as self motion signals at the top level (currently assumed to be stationary).

Also, the dots motion speed is unusually small (1/s): is there a specific reason?

The speed was chosen based on pilot experiments that showed a clear context effect of the surround on the percept of the center motion. The effect appeared to become smaller at higher speeds, presumably resulting from observers perceiving the center independent of the surround (as predicted by our model where the probability of perceiving two moving elements to belong to the same causal structure decreases with an increase in the relative velocity). This has also been observed empirically in an earlier study (Chen et al. 2014) in

which the authors found highest repulsion effects at low speeds (0.5-1 degree/s) similar to our speeds. We now clarify this in the manuscript.

Added lines 426-430

This relatively slow speed was chosen to maximize effects size during initial piloting, and is consistent with earlier studies that found high repulsion effects at slower speeds (Chen et al. 2014). This speed dependence of effect magnitude is predicted by our model: center and surround become less likely to be causally connected at higher speeds due to the higher relative velocity.

Finally, the rationale of the initial “back and forth” motion of the stimuli in experiment 1 is not clear to me.

Inspired by the original Johanssen experiments, we included the “back and forth” motion of the stimulus with the goal to provide evidence to the observer’s brain that the center and surround dots were causally linked. We also chose real motion rather than aperture motion in Exp 1, and the “back and forth” aspect of the stimulus allowed us to show the stimulus for a total of 4.5s without the center dots crossing over the surround dots.
Exp 2 qualitatively replicated the effects we found without a “back and forth” motion, and with aperture motion rather than object motion (as in Exp 1) – strongly suggesting that our results are robust and general.

Added lines 433-435

The back-and-forth movement ensured that the envelopes stayed within a fixed area of the screen. The back and forth movement was also chosen to increase the evidence to the observer’s brain that the center and surround dots were causally linked.

As a matter of fact, the proposed theory is tested on motion direction rather than speed estimation: this is not a problem as the results are very meaningful for the general framework anyway, but this specificity should be acknowledged, because some differences exist between speed and direction perception (e.g. the robustness to sensory noise, and others) and could for instance affect the transition from integration to segregation in more complex displays.

Thank you for this suggestion! We have now added this point to the discussion.

Changed lines 380-382

Furthermore, since our work was entirely based on the perception of motion directions, future work should also test our model predictions for speed perception.

Role of the zero and low-speed Prior (with respect to the previously established low-speed Prior): the results and model fitting provided in this study are very convincing and clearly support the relevance of the mass associated to purely static objects in the Prior. However, this novel and interesting (and a posteriori one could even add intuitive!) result should be further discussed, in my view. For instance Stocker and Simoncelli (2006) found that motion perception data were better explained by a slowly decreasing Prior (with heavier tales than a Gaussian): isn’t their finding contradictory with respect to the present one, where a lot of mass in zero-velocity is needed? Could the different experimental contexts (fine speed

discrimination of low contrast single moving stimuli in their case; direction estimation of structured multi-dots motion here) explain why different Prior shapes could satisfactorily explain the two sets of results? Or is this after all not a relevant difference?

Thank you for the suggestion to expand the discussion relating our work to the prior work of Stocker & Simoncelli – we now added the following paragraph to our Discussion section:

Changed lines 358-365

The mixture prior over velocity that we propose extends the slow-speed prior previously proposed by Weiss et al. (2002) and quantitatively investigated by Stocker & Simoncelli (2006). Strikingly, we found that most of the prior mass was in the delta component not included in earlier work. Most likely, the delta component was not required to explain the data in Stocker & Simoncelli (2006) since the speeds used in their experiments were too large for the delta component to matter. On the other hand, their data suggested a prior shape of speeds with heavier tails than the prior used by us (our Gaussian prior over velocity implies a Raleigh distribution over speed). Future work may want to combine a delta at zero with a heavier-tailed distribution over velocity to better match data at large velocities.

Causal vs hierarchical inference for motion perception: a more expanded illustration on the different predictions (with examples of specific differences, rather than only the AIC comparison of figure 4B) between Bill et al. model vs the present one in the context of motion integration/segmentation would further strengthen the paper.

Thank you for suggesting to expand on the model predictions for the alternative models. We have now split the model fit figures into two, with the new Figure 4 containing the model predictions for all models considered in model comparison. The model predictions are presented as violin plots overlaid with data (as done in Figure 3 for the best fitting model). We have also added a detailed explanation for predictions under different hypotheses

Added Figure 4 and lines 219-234

The predicted response for each of these models is shown in Figure 4C overlaid with observer responses. Structure sampling reports the mean under the sampled structure. Thus any variability conditioned on the structure must be due to observation and motor noise which are shared across all structures (and cannot be ascribed to variability due to sampling conditioned on the structure, as for posterior sampling). This leads to larger-than-required predicted variability for some center directions (seen prominently in observer 3), reducing the likelihood of the data under this model.
Model averaging which reports the weighted mean across structures, increases this problem since averaging reduces variability. Furthermore, it also predicts intermediate reports which struggle to explain bimodal features in the data (seen prominently in observer 1). Model selection reports the posterior mean within the structure with the highest posterior probability. While able to explain bimodal reports due to observation noise varying which structure is most likely, it overestimates observation and motor noise for the same reason that structure sampling and model averaging do: the low variability of the mean velocity conditioned on the structure (seen prominently in observer 1).
Due to the absence of the delta prior in the mixture-prior-absent hypothesis, the predicted responses show weak segmentation, and increased observation noise in order to capture

the increased variability in the data during the transition (the model fit for observer 1 tried to attribute the responses around 0 to a constant lapse, hence showing some bimodal signatures).

Figure 4.C-F : the illustration of the different structures is confusing, in particular I find that there are too many arrows and the role of each graphic element in the figures is not explained either in the caption or in the main text and it is actually not intuitive at all. I recommend simplifying the graphics, or, alternatively, to explain the match between the graphic plots and the theoretical structures more in detail in the text (but I would favor the first option, to simplify).

Thank you for your suggestion. We tried to simplify the graphics by replacing the arrows with the variable names, but that cluttered the graphics more. So we decided to retain the figure but split it and moved it to another section to separate it from the model comparison figure. Hopefully, this makes the structures easier to follow. We would happy to consider any specific suggestions that the reviewer may have.

Discussion, Lines 303-308: the reasoning in this paragraph is not clear and possibly misleading: the cited study by Chopin and colleagues focuses on binocular rivalry with perceptually biased stimuli and it seems somewhat risky to draw a parallel between the fusion/rivalry phenomenon there and the motion integration/segregation here. Similarly, the manipulation of the serial effects considered in the study by Cicchini and collaborators do not have a straightforward correspondence with the manipulation in this manuscript. Overall, I would say that the context of those experiments and the present one is not comparable at this stage, or not in an obvious way: in order to make the comparison more plausible one could consider different manipulations of the perceived speed of the surround dots (e.g. obtained by lowering their contrast or another extrinsic experimental variable)… Finally, I do not really see how the arguments discussed here would support two different models of inference for perception along the dorsal and ventral streams. This claim seems far-fetched, and I would urge the authors to provide in-depth and clear arguments in favor of this conclusion, or to omit it.

An earlier hypothesis that we had entertained, and rejected using Experiment 2 in our manuscript, is that motion is perceived relative to the perceived motion of a reference frame, not the actual (physical) motion of that reference frame. We saw this as a contrast to the finding by Cicchini et al. that in the surround tilt illusion “Maximal serial dependence for a neutral stimulus preceded by an illusory one occurred when the perceived, not the physical, orientations of the two stimuli matched” (Cicchini et al. 2020).

However, this point is not crucial to our paper so in order to avoid any confusion or misunderstanding we have now removed the corresponding paragraph from the Discussion section.

Minor points:

- The seminal paper by Ernst and Banks (2002) is cited sometimes in a not fully appropriate context: at line 115 for instance a reference to a more general work about the combination of independent noisy sensory cues would be more appropriate (e.g. the book “Sensory cue integration”, Oxford University Press, or even an earlier reference among the theoretical studies by Youille, or similar ones…)

Changed lines 125-128

Furthermore, since the observed velocities for each dot will slightly deviate from each other due to observation noise, the group velocity combines both observations to obtain a more reliable velocity percept of the group (Trommershauser et al., 2011).

- Discussion, Lines 298-302 (starting with “A corollary to this result…”): this paragraph is not clear at all to me. It is maybe only my problem, but I guess that it should be more explicit about the logical step

Under certain assumptions there is a direct relationship between the magnitude of the integration bias in structure 1, and the segmentation bias in structure 4 – consistent with the effect seen in Wu and Spering 2022. However, while unpacking this point we realized that these assumption are actually less likely to be true, and the relationship more complex, than we originally thought, so we decided to remove this statement.

- I spotted few small typos here and there:
Caption of Figure 5, “h” missiong in “has”
Line 278, “our our”
Line 377, symbol of degree missing
Line 416, “s” missing
Line 461, “n” missing

We thank the reviewer for their comments and have incorporated these into the revised manuscript.

We thank the reviewers for their positive evaluation and provide a point-by-point response to the remaining comments of Reviewer 1. We also quote the changes made in the manuscript in red.

Reviewer #1 (Remarks to the Author):

The authors have done a good job addressing my comments. I have a few easily addressed comments about the discussion of past literature:

Thank you for your comments and your attention to detail! We definitely want to get things right about the past literature.

"use priors that are not justified by our knowledge about the world but instead by computational tractability (Jäkel et al., 2016)". I don't see how this statement how this statement is valid. First, the model in that paper was indeed motivated by a claim that hierarchical reference frames reflect 'knowledge of the world' imputed to human observers. Second, I think the authors are referring to the fact that in the 2016 paper a Gaussian process assumption was invoked, which has convenient analytical properties (under a superposition assumption, the latent functions can be marginalized). This assumption could be criticized (indeed the original authors criticized it themselves), but it was not made *purely* for computational convenience. This was a substantive hypothesis about how humans analyze hierarchical motion structure, and it provided a good qualitative and quantitative match to the available data. I don't think it's necessary to take vague pot-shots at past work in order to motivate the current work. [Incidentally, I think maybe the authors meant to cite Gershman et al., 2016 here?]

We definitely did not want to take any vague pot-shots at prior work, especially not the seminal Gershman et al. 2016 paper (which is what we wanted to cite, thank you for pointing the typo!) which greatly inspired our work. What we wanted to express is that the choice of a Chinese restaurant process was motivated by mathematical convenience as a prior over structures, and contrast it with the mixture prior motivated by friction used by us. We have now clarified this point, making explicit that the hierarchical nature of the prior used by Gershman et al. reflects the structure of the physical world, and that our comment only referred to the specific formalization as a Chinese restaurant process with unclear relationship to the physical world.

Changed lines 23-26

relied on a hierarchical prior that was inspired by the nested structure of the physical world but formalized as a two-parameter hierarchical Chinese restaurant process for computational convenience (Gershman et al. 2016), making it uncertain whether this formalization can adequately capture the complexity of real-world physics or be clearly interpreted in physical terms.

Explicit questions about structure identity used in past work are criticized as being prone to decision biases. While I understand this is possible in principle, I think it's encumbent on the authors, if they really want to make this point, to show how previous results could have arisen artifactually from decision biases.

We’re not doubting or criticizing previous results, just pointing out potential problems. We have now eliminated this criticism.

"Other studies (Bill et al. 2020) employ a motion prediction task with reports in the retinotopic reference frame. This design avoids the problem of decision biases by inferring the structure from reports about a different question. However, while successful in identifying the structure, this design does not address how the motion is perceived given the inferred structures." I don't understand this. Isn't the motion prediction task precisely getting at how motion is perceived given the inferred structures?

The complication that we’re pointing out is that a prediction in retinal coordinate system cannot tell us about the reference frame underlying our perception, and hence perceived velocity. Here’s how we now phrase it in the revised manuscript:

Changed lines 26-32

Furthermore, quantitative empirical tests of some motion models have relied on explicit questions about structure identity (e.g., “Do you perceive structure A or B?” (Gershman et al. 2016, Yang et al. 2021). Other studies (Bill et al. 2020) employ a motion prediction task in an experimenter-defined retinotopic reference frame which may differ from the reference frame underlying observer percepts.
In both cases, these designs do not directly probe the velocity observers actually perceive, because they focus on either structure judgments or predictions within an experimenter-defined coordinate system rather than on perceived motion itself.

The discussion of sampling vs. MAP reports was confusing to me, because Bill et al. (2020) assumed MAP inference over structure and then sampling of perceptual reports (this might count as a 'flexible' mapping). It's important to distinguish sampling for structure inference and sampling for perceptual reports.

We agree that MAP inference over structures and MAP within a structure is different from MAP inference over structures and sampling within a structure. We have now clarified this point

Changed lines 342-344

Our data strongly constrained this mapping to correspond to sampling over both structures and velocities within each structure, different from the MAP inference over structures assumed in related prior work
